# Bayesian modelling of phosphorus content in wheat grain using hyperspectral reflectance data

**DOI:** 10.1186/s13007-023-00980-9

**Published:** 2023-01-20

**Authors:** Rosa Angela Pacheco-Gil, Ciro Velasco-Cruz, Paulino Pérez-Rodríguez, Juan Burgueño, Sergio Pérez-Elizalde, Francelino Rodrigues, Ivan Ortiz-Monasterio, David Hebert del Valle-Paniagua, Fernando Toledo

**Affiliations:** 1 Biometrics and Statistics Unit, International Maize and Wheat Improvement Center (CIMMYT), Texcoco, México; 2 Integrated Development Program, International Maize and Wheat Improvement Center (CIMMYT), Texcoco, México; 3 Socioeconomics, Statistics and Informatics Department, Colegio de Postgraduados, Texcoco, México; 4 Lincoln Agritech Ltd, Lincoln University, Canterbury, New Zealand

**Keywords:** Bayesian statistics, Hyperspectral reflectance, Wheat, Spatial analysis, Wavelength, Phosphorus

## Abstract

**Background:**

As a result of the technological progress, the use of sensors for crop survey has substantially increased, generating valuable information for modelling agricultural data. Plant spectroscopy jointly with statistical modeling can potentially help to assess certain chemical components of interest present in plants, which may be laborious and expensive to obtain by direct measurements. In this research, the phosphorus content in wheat grain is modeled using reflectance information measured by a hyperspectral sensor at different wavelengths. A Bayesian procedure for selecting variables was used to identify the set of the most important spectral bands. Additionally, three different models were evaluated: the first model assumes that the observations are independent, the other two models assume that the observations are spatially correlated: one of the proposed models, assumes spatial dependence using a Conditionally Autoregressive Model (CAR), and the other through an exponential correlogram. The goodness of fit of the models was evaluated by means of the Deviance Information Criterion, and the predictive power is evaluated using cross validation.

**Results:**

We have found that CAR was the model that best fits and predicts the data. Additionally, the selection variable procedure in the CAR model reveals which wavelengths in the range of 500–690 nm are the most important. Comparing the vegetative indices with the CAR model, it was observed that the average correlation of the CAR model exceeded that of the vegetative indices by 23.26%, − 1.2% and 22.78% for the year 2010, 2011 and 2012 respectively; therefore, the use of the proposed methodology outperformed the vegetative indices in prediction.

**Conclusions:**

The proposal to predict the phosphorus content in wheat grain using Bayesian approach, reflect with the results as a good alternative.

**Supplementary Information:**

The online version contains supplementary material available at 10.1186/s13007-023-00980-9.

## Background

Wheat, like all plants, requires nutrients and macro nutrients for its development. A balanced contribution of these, leads to good grain yield and a good quality product. Phosphorus is a nutrient which is as a source of energy necessary for all metabolic processes in the wheat plant to take place. Its deficiency makes it impossible for the plant to complete these metabolic processes normally. The two critical moments in which its presence is fundamental are: (1) in germination because it favors rapid root growth, and (2) in pre-flowering and growing because it provides the necessary energy for both grain synthesis and transport of photosynthesized sugars [1]. The phosphorus content in wheat grain, in addition to impacting the performance behavior, represents a product with a high nutritional content [1]. The determination of phosphorus content by using traditional methods is expensive and time consuming, therefore new methods to estimate this content in more efficient ways are needed.

Different vegetative indices have been used to indirectly measure the phosphorus content in wheat grain, such as the Simple Ratio [4], Normalized difference vegetation index [4], Green normalized difference vegetation index [11], Soil adjusted vegetation index [12] and Optimized soil adjusted vegetation index [19], however, these indices have been developed for monitoring N in plants.

A vegetative index that monitors phosphorus in the wheat plant is P_1808_1460 [13], for which it is necessary to adjust the reflectance data to obtain values of the complete light spectrum including the shortwave infrared region (SWIR), we can’t compare our results with this vegetative index because there was no information on this light spectrum.

We can also find studies to estimate phosphorus using reflectance data in other crops, such as [17], where they used neural networks to ascertain the key wavelengths for phosphorus prediction in savanna grass; but nothing specific for predict the phosphorus content in wheat grain, for that reason this research proposes a method to estimate it using hyperspectral reflectance.

We propose to evaluate three models: Model 1 assumes that the observations are independent, the other two models assume that the observations are spatially correlated, following either modeled by an exponential correlogram or using a Conditionally Autoregressive Model (CAR). In all models, to satisfy the assumptions of normality the dependent variable is the natural logarithm of the phosphorus concentration in the wheat grain; the independent variables are the wavelength bands measured in nanometers.

The organization of the article is as follows. In material and methods section, we describe a real dataset used for studied the predictive ability of 3 different models and to be able to compare the results with different vegetation indices. We describe the methodology and criteria for select variables, and how we did the cross-validation and predictions in each model. Next, we describe a simulation experiment to show that the proposed models work. Finally, we present the results and discussion. We include an appendix on the derivation of the full conditional distributions necessary to implement Gibbs sampler and Metropolis-Hastings algorithms and the supplementary material includes R codes that implement the proposed algorithms.

## Materials and methods

### Field experiment and data acquisition

An experiment was carried out at the International Maize and Wheat Improvement Center (CIMMYT), located in the Yaqui valley near to Obregon, Sonora, in the northwest from Mexico. The experiment aimed to investigate the effect of different levels of phosphorus (P) fertilization on P content in the wheat grain. No fertilization was performed with P in the experimental area during the previous four wheat cycles of the experiment to avoid residual effect. The climate in the Yaqui valley is semi-arid with variable precipitation rates averaging 280 mm per year and an average daily temperature of 24 °C. The soils in this region are clay coarse sandy, mixed with montmorillonite clay.

An experiment in split plots to evaluate the phosphorus in wheat grain was carried out in 3 cycles corresponding to the years 2010, 2011 and 2012. Two levels of phosphorus, 0 kg/ha and 80 kg/ ha, were considered in the main plots and 21 different wheat genotypes in subplots. 3 repetitions were performed. Each of the 126 plots, was 4 beds of 0.8 m with two rows on the top by 5 m long.

Hyperspectral reflectance was measured in each plot using a JAZ spectroradiometer Z31 with a CC-3 cosine corrector attached to the optical fiber with a FOV (field of view) aperture of 25° (Ocean Optics, Dunedin, FL, EE.UU.). The sensor has a spectral range of 339 to 1029 nm (nm) with a bandwidth of 0.38 nm, giving a total of 2048 bands. A dark reading was taken just before measurements to set a lower reflectance point of the device. A diffuse white reflectance target, Spectralon (Labsphere, North Sutton, NH, EE.UU.) was used from time to time for field measurements as reference for the upper reflectance point of the device. The data was downloaded and subsequently calibrated using SpectraSuite software (Ocean Optics, Dunedin, FL, EE.UU.). Measurements were taken at the center of each of the four beds, typically from 11:00 am. to 2:00 p.m., targeting at the canopy at a constant height. This procedure was done 2 weeks after anthesis.

**Fig. 1:**
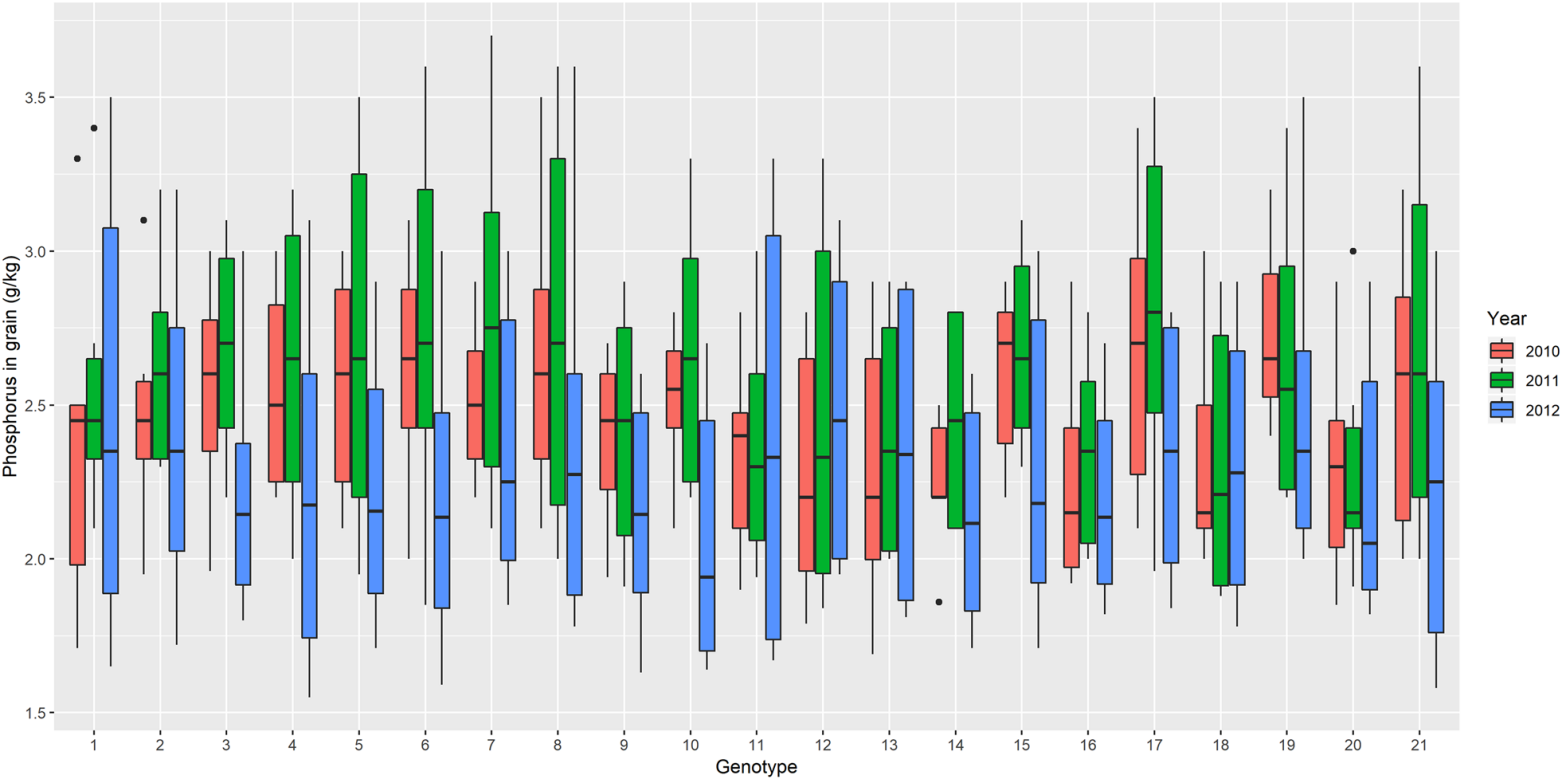
Boxplot of wheat grain phosphorus in each of the 21 genotypes for each of the 3 years

At the start this experiment was carried out for purposes of fertilization studies, but for our purpose only the data was retrieved because there were 21 wheat genotypes in 2010 and 2011, in addition to another 21 in 2021, this would allow us to capture the variability not only between individuals but also the spatial one and be able to compare the results under 3 scenarios in the sense that it can be observed variability in each year (Fig. 1) and it is due to this capability of capture the variability that predictions can improve or worsen.

Figure 1 shows the distribution of phosphorus content in wheat grain in the genotypes of the years 2010, 2011 and 2012. In 2012, 21 different genotypes were included than in the previous years and it is generally observed that these genotypes have lower phosphorus content in the grain.

### Pre-processing of hyperspectral reflectance data

Information about 2048 spectral bands was available for use as independent variables in the models. Nevertheless, it is observed that there is up to 65% of data lost in both the first 296 bands and the last 592 bands, so we decided to narrow the range of light spectrum, considering only the range from 450 to 850 nm. Additionally, it is known from several studies that multicollinearity exists between the bands of the spectrum and in our case the information was available with a narrowband of 0.38 nm bandwidth, with which linear combination in parts could be generated to make a resampling using Bsplines [7] and stay with a bandwidth of 4 nm, resulting in 101 total wavelengths used for the data analysis.

### Models

Consider the model:

1 $${y}_{i}={x}_{i}^{t}\beta +{\epsilon }_{i}$$

where ${y}_{i}$ is the natural logarithm of phosphorus in wheat grain in case $i=1,\dots ,n$, ${x}_{i}^{t}={(x}_{i1},\dots ,{x}_{ip})$ represents the reflectance of light value in each wavelength and ${\epsilon }_{i}|{\sigma }^{2}\sim NIID\left(0,{\sigma }^{2}\right)$ where “NIID” stands for normal independent and identically distributed random variables, $\beta$ is the vector of effects for each of the independent variables and ${\sigma }^{2}$ is the variance component associated to the residuals.

Model (1) can be further extended to include spatial correlation between observations, the so called geo-spatial model which can be written as:

2 $${y}_{i}={x}_{i}^{t}\beta +{w}_{i}+{\epsilon }_{i}$$

where $w = \left( {w_{1} , \ldots ,w_{n} } \right)\prime$ is the spatial random effects vector with distribution $N\left(0,{\sigma }^{2}\mathrm{H}\left(\phi \right)\right)$ and ${\tau }^{2}$ is the variance component of $y$, $\mathrm{H}\left(\phi \right)=exp\left(-\phi ||{s}_{i}-{s}_{j}||\right)$, is the isotropic correlation function, where $||{s}_{i}-{s}_{j}||$ is the Euclidean distance between the site *i* and *j* [2].

Another model used frequently in spatial statistics for dealing with aerial data is CAR, the model can be written as:

3 $${y}_{i}|{y}_{j:i\ne j}={x}_{i}^{t}\beta +{\sum }_{j=1}^{n}{c}_{ij}\left({y}_{j}-{x}_{j}^{t}\beta \right)+{\epsilon }_{i}$$

where ${\epsilon }_{i}|{\tau }^{2}\sim NIID\left(0, {\tau }^{2}\right)$, ${\tau }^{2}>0$ and ${c}_{ij}>0$ are covariance parameters, with ${c}_{ii}=0$ for all *i*. For the set of full conditional distributions to determine a well-defined joint distribution for $y$ we need to consider:

$$y\sim N\left(X\beta , {\tau }^{2}{\left({D}_{M}-\phi M\right)}^{-1}\right)$$

where ${\tau }^{2}$ is the variance component of $y$, $M$ the neighborhood matrix, ${D}_{M}={\sum }_{j=1}^{n}{m}_{ij}$ and $\phi$ is the autocorrelation parameter related to the ordered eigenvalues $\left({\lambda }_{(1)}<{\lambda }_{(2)}<\dots <{\lambda }_{(n)}\right)$ of ${{D}_{M}}^{1/2}M{{D}_{M}}^{1/2}$ (for see more details consult [2]).

### Selection Criteria

Intrinsic to the adjustment, the method includes the selection of bands (variables), by adapting the method proposed by [10], which induces variable selection. As a result, the posterior probability is obtained, $p\left({\beta }_{j}\ne 0|data\right)$, where ${\beta }_{j}$ is the regression coefficient corresponding to the *j*-th band. Or similarly, $p\left({\gamma }_{j}=1|data\right)$, where ${\gamma }_{j}$ is an indicator variable corresponding to the *j*-th band, which is equal to 1 if ${\beta }_{j}\ne 0$, and 0 otherwise. With this information the spectral bands can be selected under two criteria:

Those with $$p\left({\beta }_{j}\ne 0|data\right)\ge$$ 0.6 [3].

The *γ* vector represents a submodel, its probability was obtained by counting the frequency of each submodel, in this way we can classify the submodels and identify which one is the most likely (avgmod).

Once the previous criteria for band selection have been applied and their respective parameters have been estimated in each model, the Deviance Information Criterion (DIC) [20] is calculated in order to select the best model.

### Sampling model and likelihood function

Assuming a random sample from (1), (2) and (3) respectively. Then the conditional distribution for (1) ${y}_{i}|\beta ,{\sigma }^{2}$ is normal with mean ${x}_{i}^{t}\beta$ and variance ${\sigma }^{2}$. The conditional distribution for (2) ${y}_{i}|\beta ,{\sigma }^{2},{\tau }^{2},\phi ,w$ is normal with mean ${x}_{i}^{t}\beta +{w}_{i}$ with variance ${\tau }^{2}$. Finally, conditional distribution for (3) ${y}_{i}|\beta ,{\tau }^{2},\phi$ is normal with mean ${x}_{i}^{t}\beta$ and variance ${\tau }^{2}{\left({\sum }_{j=1}^{n}{m}_{ij}-\phi {m}_{i.}\right)}^{-1}$. In general, we the joint conditional distribution of $y|\theta$ is given by $p\left(y|\theta \right)=\prod_{i=1}^{n}p\left({y}_{i}|\theta \right)$, where $\theta$ denote the model unknowns for each model.

### Prior distributions

In order to complete the specification of the models, we assign prior distribution to the model unknowns. Let $p\left({\beta }_{j}|{\gamma }_{j}\right)=\left(1-{\gamma }_{j}\right){p}_{spike}\left({\beta }_{j}\right)+{\gamma }_{j}{p}_{slab}\left({\beta }_{j}\right)$, with ${p(\gamma }_{j}=0)={p}_{j}$, ${p}_{spike}\left({\beta }_{j}\right)={I}_{(0)}{(\beta }_{j})$ and ${p}_{slab}\left({\beta }_{j}\right)=N({\beta }_{j}|{\mu }_{j},{\nu }_{j})$ for each model. For model (1) ${\sigma }^{2}|{\alpha }_{11},{\eta }_{11}\sim IG({\alpha }_{11},{\eta }_{11})$. In model (2) ${\sigma }^{2}|{\alpha }_{21},{\eta }_{21}\sim IG\left({\alpha }_{21},{\eta }_{21}\right),$ ${\tau }^{2}|{\alpha }_{22},{\eta }_{22}\sim IG({\alpha }_{22},{\eta }_{22})$ and for $\phi >0, \phi |min,max\sim U(min,\mathrm{max})$. And for model (3) ${\tau }^{2}|{\alpha }_{31},{\eta }_{31}\sim IG({\alpha }_{31},{\eta }_{31})$ and $\phi |\frac{1}{{\lambda }_{(1)}}, \frac{1}{{\lambda }_{(n)}}\sim U\left(\frac{1}{{\lambda }_{(n)}}, \frac{1}{{\lambda }_{(1)}}\right)$. With $IG(\alpha ,\eta )$ we denote an inverse gamma distribution, whose probability density function is $f\left(x;\alpha ,\eta \right)=\frac{{\eta }^{\alpha }}{\Gamma (\alpha )}{\left(1/x\right)}^{\alpha +1}exp\left(-\eta /x\right)$ where $\alpha$ and $\eta$ correspond to the shape and rate parameters, respectively. The joint priori distribution $p\left(\theta |H\right)$ of each model unknows is given by:

$p\left( {\beta ,\sigma^{2} {|}H_{1} } \right) \propto p(\beta |\gamma )p\left( {\sigma^{2} |\alpha_{11} ,\eta_{11} } \right)$ for model (1), where ${H}_{1}=\left\{{\alpha }_{11},{\eta }_{11}\right\}$ is the set of hyper-parameters.

$p\left( {\beta ,\sigma^{2} ,\tau^{2} ,\phi ,w{|}H_{2} } \right) \propto p(\beta |\gamma )p\left( {\sigma^{2} |\alpha_{21} ,\eta_{21} } \right)p\left( {\tau^{2} |\alpha_{22} ,\eta_{22} } \right)p\left( {\phi |min,max} \right)p\left( {w|\sigma^{2} ,\phi } \right)$ for model (2), where ${H}_{2}=\left\{{\alpha }_{21},{\eta }_{21},{\alpha }_{22},{\eta }_{22},min,max\right\}$ is the set of hyper-parameters.

$p\left( {\beta ,\tau^{2} ,\phi {|}H_{3} } \right) \propto p(\beta |\gamma )p\left( {\tau^{2} |\alpha_{31} ,\eta_{31} } \right)p\left( {\phi |\frac{1}{{\lambda_{\left( 1 \right)} }},{ }\frac{1}{{\lambda_{\left( n \right)} }}} \right)$ for model (3), where ${H}_{3}=\left\{{\alpha }_{31},{\eta }_{31},\frac{1}{{\lambda }_{(1)}}, \frac{1}{{\lambda }_{(n)}}\right\}$ is the set of hyper-parameters.

### Posterior distributions

The joint posterior distribution of all quantities can be obtained by applying the Bayes’ theorem, so we obtain $p\left(\theta |data,H\right)\propto p\left(y|\theta \right)p\left(\theta |H\right)$.

The hierarchical structure of this distribution allows us to obtain the conditional distributions necessary to implement the Gibbs sampler [8] and draw samples from the joint posterior distribution, in other form this distribution is analytically un-tractable. Not all full conditional distributions have a closed form, for that reason was necessary to implement the Metropolis–Hastings algorithm [6]; the algorithms are described in the Appendix.

The hyper-parameters for the inverse gamma distributions are set as $\alpha =\eta =0.01$ because it provides a weakly informative prior [14]; for the uniform distributions are set as $min$ =0, $max=1$, and ${\lambda }_{(1)},{\lambda }_{(n)}$ are the eigenvalues mentioned in model (3).

### Software

The algorithm to fit models was implemented in a program written in R [18]. The input arguments are the response vector y, the matrices X,w,M, the number of Markov Chain Monte Carlo (MCMC) iterations, a burn-in period and the hyper-parameters. The outputs provide the mean of the predictive distribution obtained through the MCMC algorithm and provides us with the variables selected under the selection criteria previously described, it also computes the DIC [20] and it provides the correlations that are obtained when making the cross validations. Three libraries from R were used, MCMCpack [15], mvtnorm [9] and truncnorm [16].

### Simulations

To evaluate the behavior of the variable selection procedure in each model, a simulation experiment was carried out, this consisted of generating $X$ with 100 random variables from a normal standard distribution, based on linear combinations ${x}_{i}+{x}_{j}={x}_{k}$ with $i\in \left\{\mathrm{1,3},5,\dots ,99\right\}$, $j\in \left\{\mathrm{2,4},6,\dots ,100\right\}$ and $k\in \left\{\mathrm{101,102},\dots ,150\right\}$ were generated 50 more variables, this to simulate the multicollinearity expected in real dataset. The neighborhood matrix was the same as in the real dataset. $\beta$ with values close to zero was also generated to specify variables with little effect and large values for the most important variables, let $A=\{\mathrm{2,6},\mathrm{13,33,25,67,71,77,85,94,96,99}\}$ and $B=\{\mathrm{1,2},3,\dots ,150\}$ then ${\beta }_{j}=0.9$ with $j\in A$, ${\beta }_{j}=0.01$ with $j\in B-A$; and values were set for the parameters ${\sigma }^{2}=0.3,{\tau }^{2}=0.45,\phi =0.21$. Using those data and conditional distribution we simulated $y$ for each model. Finally, we used the simulated data as an input in our R script in order to check that in each model the desired parameters are being correctly estimated.

### Cross validation and predictions

In order to have a reference regarding the predictive power of the models used, cross validation was performed. The response vector, y, was divided into 2 disjoint sets, randomly, always considering 25 observations for the testing data set and 101 for the training set. In such a way that $y=$ $\left( {y_{training} , y_{testing} } \right)\prime$. Also, the matrix of covariates or bands, was divided in a way that corresponded to the values of y, such as $X = \left( {X_{training} , X_{testing} } \right)\prime$ The models were fitted with the information corresponding to the “training”, and the adjusted model was used to predict the response of the “testing” set. This procedure was repeated 5 times.

## Results

The results presented below are derived from 100,000 iterations. The first 50,000 were discarded, to ensure convergence (which was validated graphically using the trace plots). As a sample, one in 5 of the last 50,000 were considered, with which the averages of each parameter involved, called a point estimate, were calculated. For example, for the model without spatial correlation, the point estimation of the parameters is denoted as $\left(\widehat{\beta }, {\widehat{\sigma }}^{2}\right)$. For the CAR model, the point estimation of the parameters is denoted as $\left(\widehat{\beta }, {\widehat{\tau }}^{2},\widehat{\phi }\right)$, and $\left(\widehat{\beta }, {\widehat{\sigma }}^{2}, {\widehat{\tau }}^{2},\widehat{\phi }\right)$ for the geospatial model. The DIC was calculated for each model. $p\left({\beta }_{j}\ne 0|data\right)$ was calculated as a measure of importance of each band and the most probable models, and as a measure of the predictive power of the models the Pearson’s correlation between ${y}_{testing}$ and ${\widehat{y}}_{testing}$ (the prediction of response variable based on the model adjusted with the information in the “training” set) of cross validation.

**Fig. 2:**
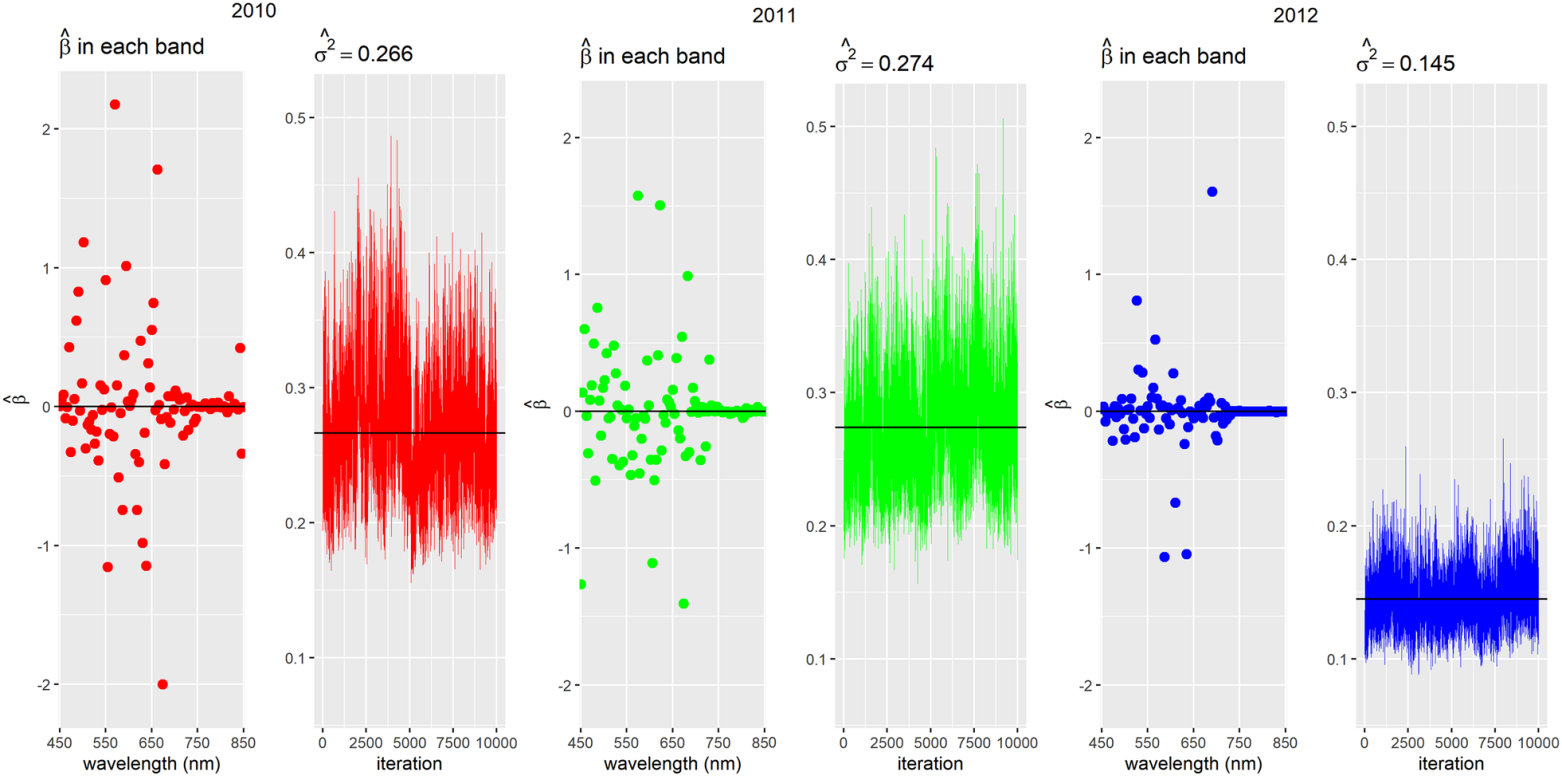
Trace plots and posterior means of estimated parameters in the model without spatial correlation per year

**Fig. 3:**
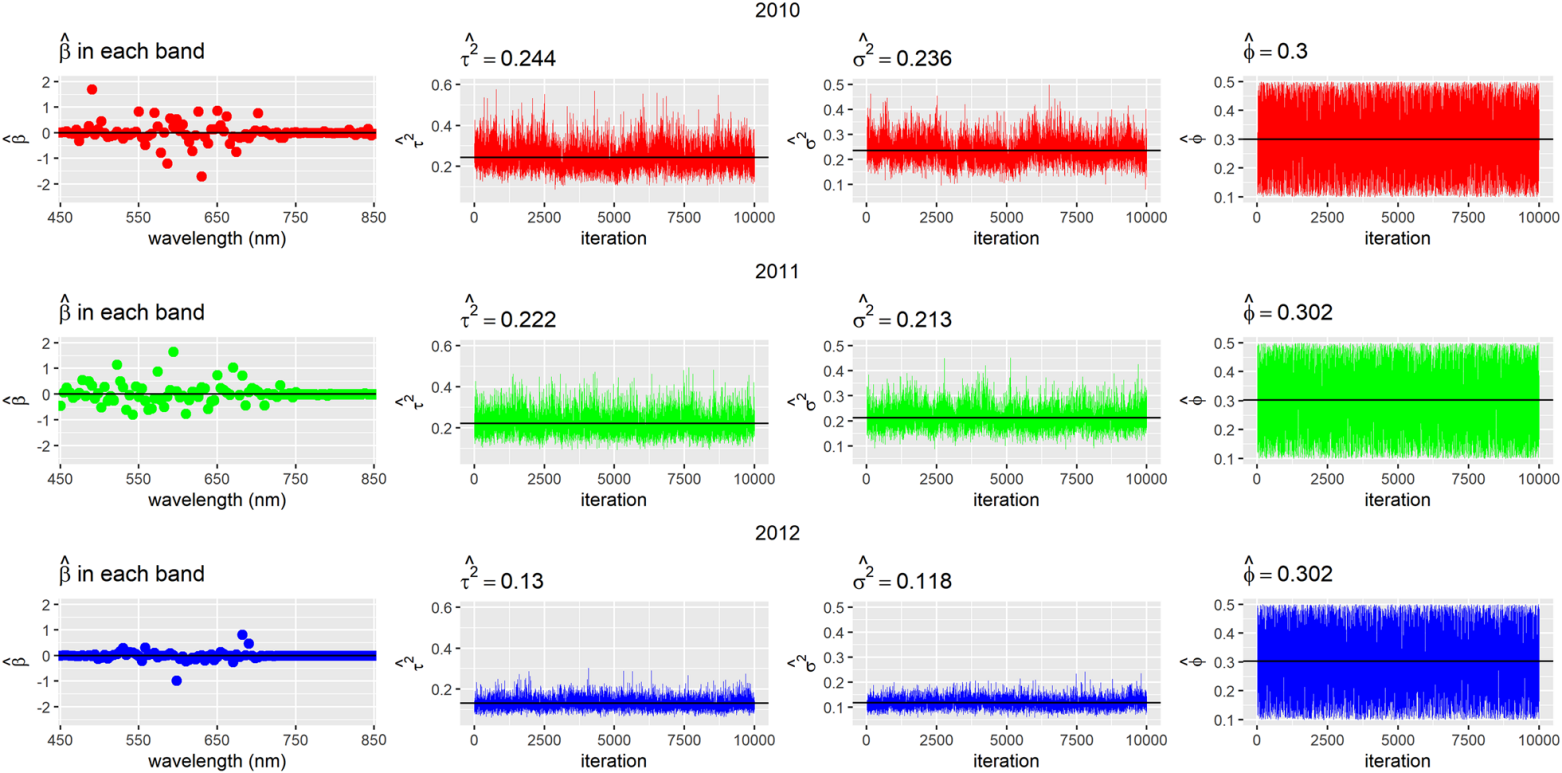
Trace plots and posterior means for parameters in the geospatial model by year

**Fig. 4:**
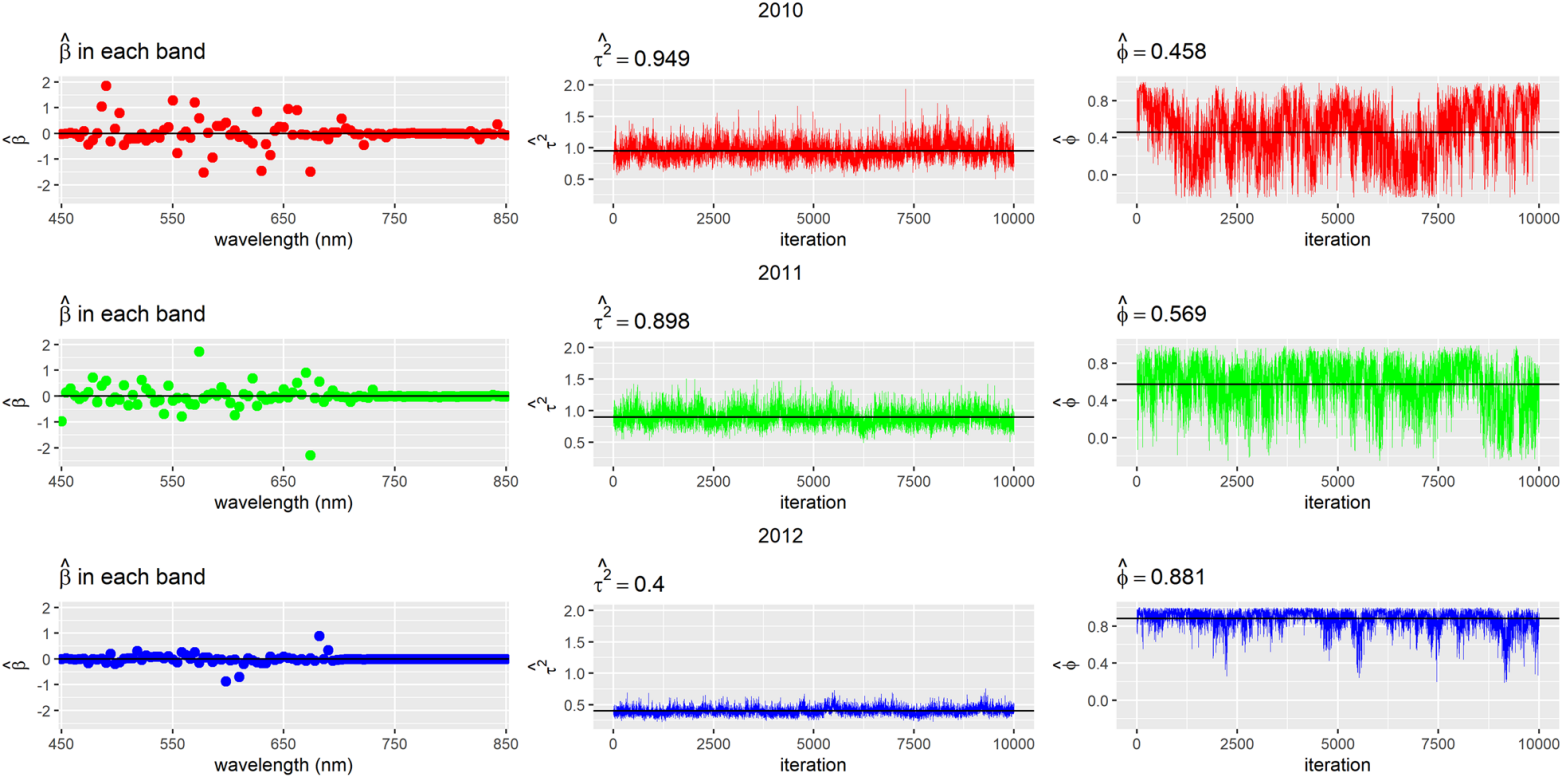
Trace plots and posterior means of parameters in the CAR model per year

In Figs. 2, 3, 4, we show the trace plots and posterior means of parameters of interest for each year when considering all wavelengths for models (1), (2) and (3). Note that the variability of the data is very similar between 2010 and 2011 (see ${\widehat{\sigma }}^{2}$ in the case of the model without spatial correlation and ${\widehat{\tau }}^{2}$ in the CAR). The 2012 variance is smaller than in the previous years. This difference could be attributed to the fact that different genotypes were used for the experiment in this last year.

With respect to the parameter of spatial variation $\left(\widehat{\phi }\right)$ in the CAR model, values were found around and above 0.5, depending on the year, which indicates a positive dependence between the plots studied. In the case of the geospatial model it is observed that the maximum distance from which there is spatial dependence is $\widehat{\phi }$ = 0.3 and that the maximum variability in the absence of spatial dependence is ${\widehat{\sigma }}^{2}$= 0.2, approximately.

**Fig. 5:**
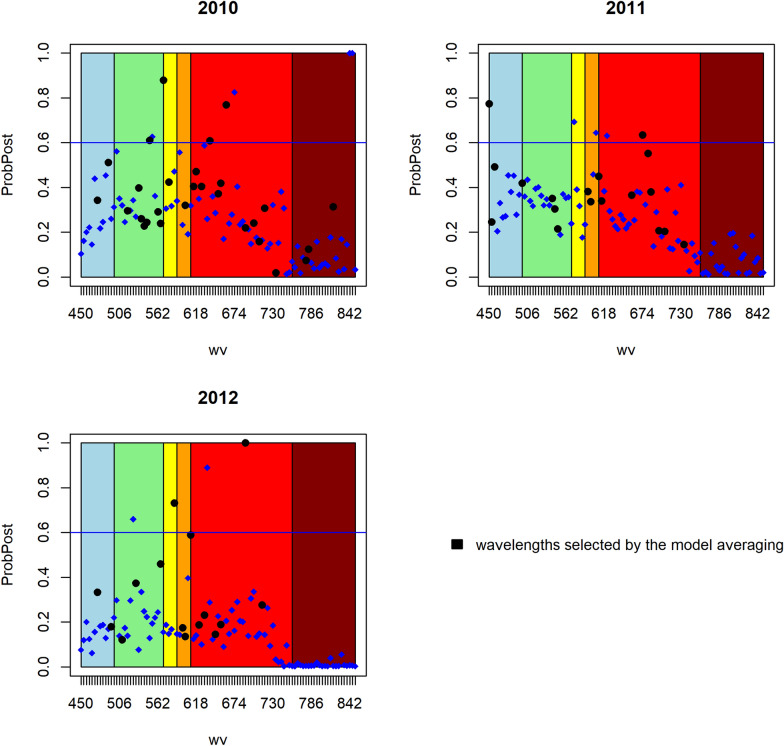
Posterior probability of the spectral bands of each year of the model without spatial correlation

**Fig. 6:**
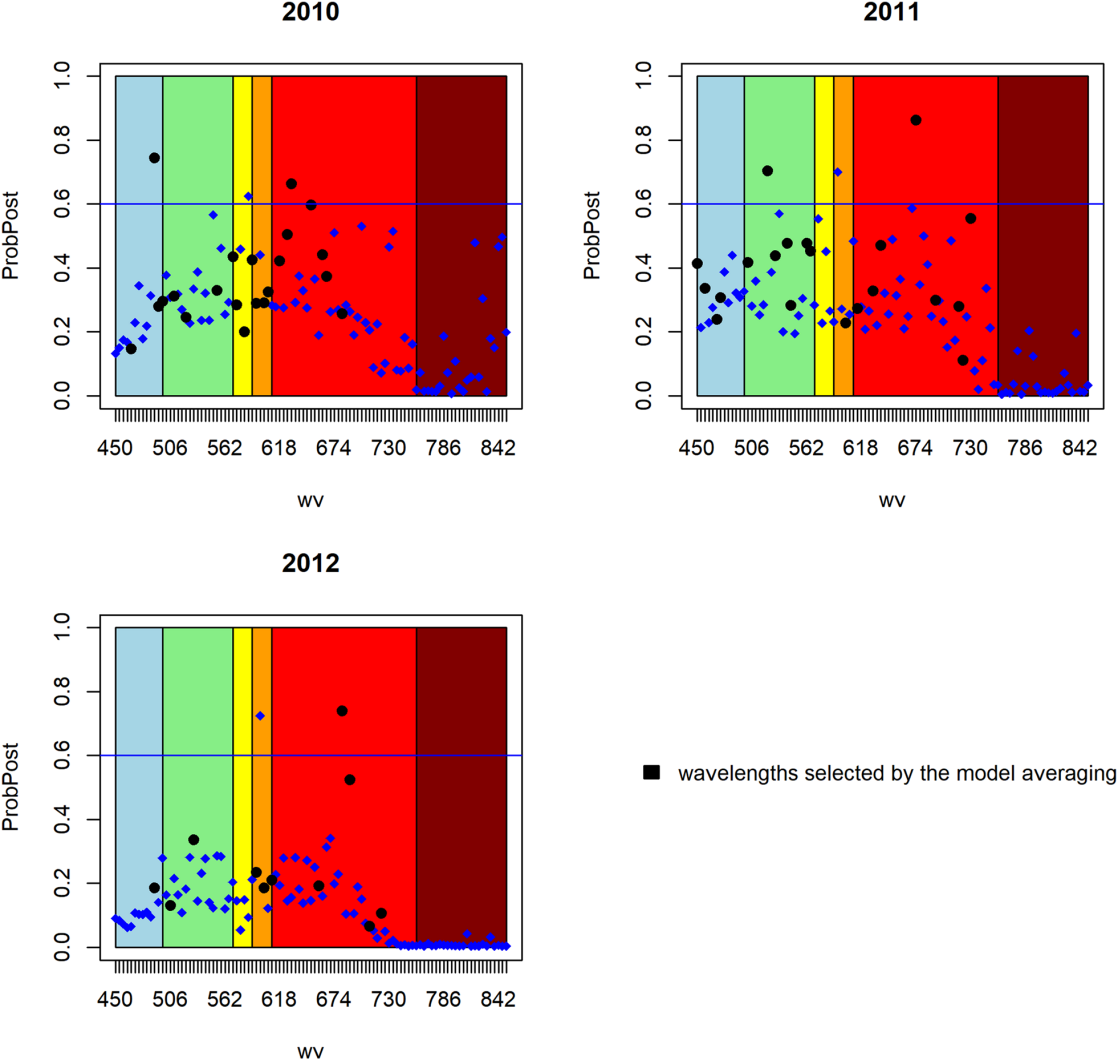
Posterior probability of the spectral bands of each year of the geospatial model

**Fig. 7:**
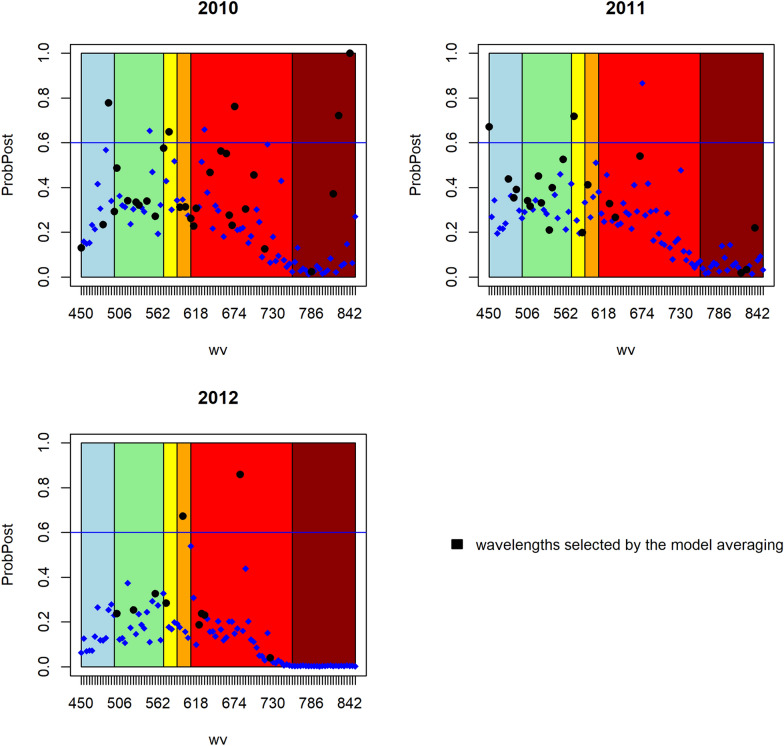
Posterior probability of the spectral bands of each year of the CAR model

In Figs. 5, 6, 7 the value of the posteriori probability for each spectral band is observed at each point, that is $p\left({\beta }_{j}\ne 0|data\right)$ graphically, the light spectrum to which they belong is also illuminated. The black dots represent a posteriori probability of the spectral bands selected by the most probable model, and above the blue horizontal line, which represents the posterior probability of 0.6, are the important spectral bands.

It can also be observed that in general the posterior probability of the spectral bands is greater in the visible spectrum from green to red, that is, in the range of 500 to 690 nm, regardless of the fitted model or the year of the experiment. However, there are no specific or recurring wavelengths that can be considered in general to directly relate them to the behavior of the phosphorus content in wheat grain.

As mentioned, the way to select the best model was through the DIC. In Table 1 it can be seen the CAR model was the selected model, since the value of the DIC obtained is the smallest for all scenarios considered and is even lower if we consider a model that includes only selected bands.

**Table 1:** DIC values

	Selection criterio								
	All bands			$p\left({\beta }_{j}\ne 0|data\right)$≥0.6			Avgmod		
Model	2010	2011	2012	2010	2011	2012	2010	2011	2012
SCE	590.67	534.12	886.98	335.74	333.37	552.68	466.71	434.38	731.89
Geospatial	438.87	461.98	288.03	485.25	314.71	548.48	542.73	392.09	493.97
CAR	242.97	226.02	121.83	254.92	233.35	162.73	239.87	211.82	102.87

To measure the predictive power of the models, cross validation was used. Table 2 shows the Pearson’s correlation coefficient calculated between ${y}_{testing}$ and ${\widehat{y}}_{testing}$ in each of the models used, for each year, for each wavelength selection. Consistently, it is observed that the CAR model on average is the one that has the best predictive power when presenting the highest correlations.

**Table 2:** Average correlations in cross-validation

	Selection criterio								
	All bands			$p\left({\beta }_{j}\ne 0|data\right)\ge$ 0.6			Avgmod		
Model	2010	2011	2012	2010	2011	2012	2010	2011	2012
SCE	0.642	0.677	0.652	0.523	0.389	0.635	0.533	0.630	0.634
Geospatial	0.647	0.736	0.749	0.499	0.639	0.630	0.595	0.671	0.733
CAR	0.655	0.709	0.814	0.524	0.621	0.647	0.601	0.680	0.823

Once the CAR model was selected to model the P content in the wheat grain, we proceeded to select from the literature some vegetative indexes that have been used to monitor the nutrient content in plants (N, P, K, S), as done in [13] and with these evaluate the prediction of the CAR model. The same 5 subsets of cross-validation tests were used to calculate the vegetation indices in Table 3 and obtain the correlation coefficient for each year. In general, for the three years, the results with the CAR model are better than with the vegetative indices, since their average correlation remains constant above 0.6 or more, rather what stands out is the fact that the vegetative indices alone cannot be trusted because is clearly observed that we can have both good results as in the case of year 2011 and 2012, but very bad in the year 2010 (Table 3).

**Table 3:** Average correlations in cross-validation of vegetative indexes

Index	Expression	Year		
		2010	2011	2012
Simple Ratio [4]	$\frac{{R}_{800-900}}{{R}_{650-700}}$	0.338	0.738	0.666
Normalized difference vegetation index [5]	$\frac{{R}_{800}-{R}_{680}}{{R}_{800}+{R}_{680}}$	0.356	0.685	0.527
Green normalized difference vegetation index [11]	$\frac{{R}_{800-900}-{R}_{540-560}}{{R}_{800-900}+{R}_{540-560}}$	0.443	0.674	0.687
Soil adjusted vegetation index [12]	$\frac{1.5 {R}_{800-900}-{R}_{650-700}}{{R}_{800-900}+{R}_{650-700}+0.5}$	0.353	0.692	0.530
Optimized soil adjusted vegetation index [19]	$\frac{{1.16R_{800} - R_{670} }}{{R_{800} + R_{670} + 0.16}}$	0.352	0.671	0.566
	CAR model avg	0.601	0.680	0.823

R corresponds to the reflectance at corresponding subscripted wavelength (nm)

## Conclusion

Under the model selection criterion, DIC, it was concluded that the best of the adjusted models was the CAR. With the result of the cross-validation, it was concluded that the model with the best predictive power coincided with the best-adjusted model, that is, the CAR.

This was expected given that the spatial dependence in a CAR model can separate and clarify the structural and functional components, with the structural ones we understand the correlation that is determined by physical proximity (close neighbors) the functional ones refer to the correlation that it is affected by dispersion, landscape characteristics and other variables of interest that are taken into account by the CAR model. These desirable characteristics, which cannot be included with the simple calculation of an index, enhance the use of the CAR model.

Also, the a posteriori probability obtained by the implementation of variable selection in all the evaluated models is observed and a repetitive pattern that would determine that with the spectral bands selected as the most probable and lead to an index built to determine the content of phosphorus in the wheat grain was not found, however, if it could be concluded that the range of the light spectrum that goes from 500 to 690 nm, is the one that most likely directly intervenes when predicting the phosphorus content in the grain. This information is useful to recommend making a good calibration of the sensor with which reflectance readings will be taken in the range found.

Comparing the vegetative indices with the CAR model, it was observed that the average correlation of the CAR model exceeded that of the vegetative indices by 23.26%, − 1.2% and 22.78% for the year 2010, 2011 and 2012 respectively; therefore, the use of the proposed methodology outperformed the vegetative indices in prediction.

Therefore, the use of this methodology is not only useful to reduce dimensionality, even when there are multicollinearity problems, but also with the posterior probabilities obtained, the importance and/or inclusion of some band in the prediction model can be decided. It is also possible to take into account the inclusion of spatial variability in the model, with which the model (1) was surpassed in prediction by up to 19%.

In this research, this methodology is proposed to predict the phosphorus content in wheat grain, reflecting with the results as a good alternative, however, if the reflectance information and the disposition of the experiment in the field are available, it is suggested to evaluate it with other variable responses of interest such as yield for example and in other crops.

## Supplementary Information

**Additional file 1:** Appendix: Conditional distributions.

**Additional file 2:** Hyperspectral data.

**Additional file 3:** R codes.

## Data Availability

The datasets used and analyzed during the current study are available from the corresponding author on reasonable request.
