# Supplementary material for: Bayesian modelling of phosphorus content in wheat grain using hyperspectral reflectance data
Source: Plant Methods. 2023 Jan 20;19:6. doi: 10.1186/s13007-023-00980-9 (PMC9854047; doi:10.1186/s13007-023-00980-9)
Supplement: Supplementary file 1 — Additional file 1: Appendix: Conditional distributions. [file 13007_2023_980_MOESM1_ESM.docx]

Appendix. Conditional distributions for models (1), (2) and (3)

a. Conditional distributions for model (1)

1.$\boldsymbol{p}\left( \boldsymbol{\sigma}^{\boldsymbol{2}} | \boldsymbol{else} \right)$

$$\begin{matrix} p\left( \sigma^{2} | else \right) & \propto& \left( 2\pi\sigma^{2} \right)^{-\frac{n}{2}}exp\left\{ -\frac{1}{2\sigma^{2}}\left( \boldsymbol{y-X\beta} \right)^{t}\boldsymbol{(y-X\beta)} \right\}\frac{{\eta_{11}}^{\alpha_{11}}}{\Gamma(\alpha_{11})}\left( 1/{\sigma^{2}} \right)^{\alpha_{11}+1}exp\left( {-\eta_{11}}/{\sigma^{2}} \right) \\ & \propto& \frac{\left\{ \eta_{11}+\frac{1}{2\sigma^{2}}\left( \boldsymbol{y-X\beta} \right)^{t}\left( \boldsymbol{y-X\beta} \right) \right\}^{\frac{n}{2}+\alpha_{11}}}{\Gamma\left( \frac{n}{2}+\alpha_{11} \right)}\left( 1/{\sigma^{2}} \right)^{\left( \frac{n}{2}+\alpha_{11} \right)+1}exp\left\{ \left( -\eta_{11}+\frac{1}{2\sigma^{2}}\left( \boldsymbol{y-X\beta} \right)^{t}\boldsymbol{(y-X\beta)} \right)/{\sigma^{2}} \right\} \\ \sigma^{2}|else & \sim& \boldsymbol{IG}\left( \frac{n}{2}+\alpha_{11},\eta_{11}+\frac{1}{2}\left( \boldsymbol{y-X\beta} \right)^{t}\boldsymbol{(y-X\beta)} \right) \end{matrix}$$

b. Conditional distributions for model (2)

1.$\boldsymbol{p}\left( \boldsymbol{\sigma}^{\boldsymbol{2}} | \boldsymbol{else} \right)$

$$\begin{matrix} p\left( \sigma^{2} | else \right) & \propto& \left( 2\pi\sigma^{2} \right)^{-\frac{n}{2}}exp\left\{ -\frac{1}{2\sigma^{2}}\boldsymbol{w}^{t}\boldsymbol{H}^{\boldsymbol{-1}}\left( \phi\right)\boldsymbol{w} \right\}\frac{{\eta_{21}}^{\alpha_{21}}}{\Gamma(\alpha_{21})}\left( 1/{\sigma^{2}} \right)^{\alpha_{21}+1}exp\left( {-\eta_{21}}/{\sigma^{2}} \right) \\ & \propto& \frac{\left\{ \eta_{21}+\frac{1}{2\sigma^{2}}\boldsymbol{w}^{t}\boldsymbol{H}^{\boldsymbol{-1}}\left( \phi\right)\boldsymbol{w} \right\}^{\frac{n}{2}+\alpha_{21}}}{\Gamma(\frac{n}{2}+\alpha_{21})}\left( 1/{\sigma^{2}} \right)^{\left( \frac{n}{2}+\alpha_{21} \right)+1}exp\left\{ \left( -\eta_{21}+\frac{1}{2\sigma^{2}}\boldsymbol{w}^{t}\boldsymbol{H}^{\boldsymbol{-1}}\left( \phi\right)\boldsymbol{w} \right)/{\sigma^{2}} \right\} \\ \sigma^{2}|else & \sim& \boldsymbol{IG}\left( \frac{n}{2}+\alpha_{21},\eta_{21}+\frac{1}{2}\boldsymbol{w}^{t}\boldsymbol{H}^{\boldsymbol{-1}}\left( \phi\right)\boldsymbol{w} \right) \end{matrix}$$

2. $\boldsymbol{p}\left( \boldsymbol{\tau}^{\boldsymbol{2}} | \boldsymbol{else} \right)$

$$\begin{matrix} p\left( \tau^{2} | else \right) & \propto& \left( 2\pi\tau^{2} \right)^{-\frac{n}{2}}exp\left\{ -\frac{1}{2\tau^{2}}\left( \boldsymbol{y-X\beta-w} \right)^{t}\boldsymbol{(y-X\beta-w)} \right\}\frac{{\eta_{22}}^{\alpha_{22}}}{\Gamma(\alpha_{22})}\left( 1/{\tau^{2}} \right)^{\alpha_{22}+1}exp\left( {-\eta_{22}}/{\tau^{2}} \right) \\ & \propto& \frac{\left\{ \eta_{22}+\frac{1}{2\tau^{2}}\left( \boldsymbol{y-X\beta-w} \right)^{t}\boldsymbol{(y-X\beta-w)} \right\}^{\frac{n}{2}+\alpha_{22}}}{\Gamma(\frac{n}{2}+\alpha_{22})}\left( 1/{\tau^{2}} \right)^{\left( \frac{n}{2}+\alpha_{22} \right)+1}exp\left\{ \left( -\eta_{22}+\frac{1}{2\tau^{2}}\left( \boldsymbol{y-X\beta-w} \right)^{t}\boldsymbol{(y-X\beta-w)} \right)/{\tau^{2}} \right\} \\ \tau^{2}|else & \sim& \boldsymbol{IG}\left( \frac{n}{2}+\alpha_{22},\eta_{22}+\frac{1}{2}\left( \boldsymbol{y-X\beta-w} \right)^{t}\boldsymbol{(y-X\beta-w)} \right) \end{matrix}$$

3. $\boldsymbol{p}\left( \boldsymbol{w} | \boldsymbol{else} \right)$

$$\begin{matrix} p\left( \boldsymbol{w} | else \right) & \propto& \left( 2\pi\tau^{2} \right)^{-\frac{n}{2}}exp\left\{ -\frac{1}{2\tau^{2}}\left( \boldsymbol{y-X\beta-w} \right)^{t}\boldsymbol{(y-X\beta-w)} \right\}\left( 2\pi\sigma^{2} \right)^{-\frac{n}{2}}exp\left\{ -\frac{1}{2\sigma^{2}}\boldsymbol{w}^{t}\boldsymbol{H}^{\boldsymbol{-1}}\left( \phi\right)\boldsymbol{w} \right\} \\ \boldsymbol{w}|else & \sim& \boldsymbol{N}\left( \left[ \frac{1}{\tau^{2}}I+\frac{1}{\sigma^{2}}\boldsymbol{H}^{\boldsymbol{-1}}\left( \phi\right) \right]\frac{1}{\tau^{2}}\left( \boldsymbol{y-X\beta} \right)\boldsymbol{,}\left[ \frac{1}{\tau^{2}}I+\frac{1}{\sigma^{2}}\boldsymbol{H}^{\boldsymbol{-1}}\left( \phi\right) \right] \right) \end{matrix}$$

4. $\boldsymbol{p}\left( \boldsymbol{\phi} | \boldsymbol{else} \right)$

$$\begin{matrix} \boldsymbol{p}\left( \boldsymbol{\phi} | \boldsymbol{else} \right) & \boldsymbol{\propto} & \left( \boldsymbol{2\pi}\boldsymbol{\sigma}^{\boldsymbol{2}} \right)^{\boldsymbol{-}\frac{\boldsymbol{n}}{\boldsymbol{2}}}\boldsymbol{exp}\left\{ \boldsymbol{-}\frac{\boldsymbol{1}}{\boldsymbol{2}\boldsymbol{\sigma}^{\boldsymbol{2}}}\boldsymbol{w}^{\boldsymbol{t}}\boldsymbol{H}^{\boldsymbol{-1}}\left( \boldsymbol{\phi} \right)\boldsymbol{w} \right\}\frac{\mathbf{1}}{\boldsymbol{max-min}} \end{matrix}$$

c. Conditional distributions for model (3)

1. $\boldsymbol{p}\left( \boldsymbol{\tau}^{\boldsymbol{2}} | \boldsymbol{else} \right)$

$$\begin{matrix} p\left( \tau^{2} | else \right) & \propto& \left( 2\pi\tau^{2} \right)^{-\frac{n}{2}}exp\left\{ -\frac{1}{2\tau^{2}}\left( \boldsymbol{y-X\beta} \right)^{t}\left( \boldsymbol{D}_{M}-\phi\boldsymbol{M} \right)\boldsymbol{(y-X\beta)} \right\}\frac{{\eta_{31}}^{\alpha_{31}}}{\Gamma(\alpha_{31})}\left( 1/{\tau^{2}} \right)^{\alpha_{31}+1}exp\left( {-\eta_{31}}/{\tau^{2}} \right) \\ & \propto& \frac{\left\{ \eta_{31}+\frac{1}{2\tau^{2}}\left( \boldsymbol{y-X\beta} \right)^{t}\left( \boldsymbol{D}_{M}-\phi\boldsymbol{M} \right)\boldsymbol{(y-X\beta)} \right\}^{\frac{n}{2}+\alpha_{31}}}{\Gamma(\frac{n}{2}+\alpha_{31})}\left( 1/{\tau^{2}} \right)^{\left( \frac{n}{2}+\alpha_{31} \right)+1}exp\left\{ \left( -\eta_{31}+\frac{1}{2\tau^{2}}\left( \boldsymbol{y-X\beta} \right)^{t}\left( \boldsymbol{D}_{M}-\phi\boldsymbol{M} \right)\boldsymbol{(y-X\beta)} \right)/{\tau^{2}} \right\} \\ \tau^{2}|else & \sim& \boldsymbol{IG}\left( \frac{n}{2}+\alpha_{31},\eta_{31}+\frac{1}{2}\left( \boldsymbol{y-X\beta} \right)^{t}\left( \boldsymbol{D}_{M}-\phi\boldsymbol{M} \right)\boldsymbol{(y-X\beta)} \right) \end{matrix}$$

**2.** $p\left( \phi| else \right)$

$$\begin{matrix} p\left( \phi| else \right) & \propto& \left( 2\pi\tau^{2} \right)^{-\frac{n}{2}}exp\left\{ -\frac{1}{2\tau^{2}}\left( \boldsymbol{y}-\boldsymbol{X\beta} \right)^{t}\left( \boldsymbol{D}_{\boldsymbol{M}}-\phi\boldsymbol{M} \right)\left( \boldsymbol{y}-\boldsymbol{X\beta} \right) \right\}\frac{\lambda_{\left( n \right)}\lambda_{\left( 1 \right)}}{\lambda_{\left( n \right)}-\lambda_{\left( 1 \right)}} \end{matrix}$$

Conditional distributions for variable selection in all models

1. The Bayes factor for the hypothesis test in which one is in favor of the model where the *j-th* element of $\boldsymbol{\beta}$ is not included even though it is different from zero, above the model where the *j-th* element of $\boldsymbol{\beta}$ is not included knowing that this is equal to zero, it can be written as:

$$\begin{matrix} \boldsymbol{BF} & \boldsymbol{=} & \frac{\boldsymbol{p}\left( \boldsymbol{z} | \boldsymbol{\beta}_{\boldsymbol{j}}\boldsymbol{\neq0} \right)}{\boldsymbol{p}\left( \boldsymbol{z} | \boldsymbol{\beta}_{\boldsymbol{j}}\boldsymbol{=0} \right)} \\ & \boldsymbol{=} & \frac{\left( \frac{\boldsymbol{s}^{\boldsymbol{2}}}{\boldsymbol{\nu}_{\boldsymbol{j}}} \right)^{\boldsymbol{1/2}}\boldsymbol{exp}\left( \boldsymbol{-}\frac{\boldsymbol{\mu}_{\boldsymbol{j}}^{\boldsymbol{2}}}{\boldsymbol{\nu}_{\boldsymbol{j}}} \right)}{\boldsymbol{exp}\left\{ \boldsymbol{-}\frac{\boldsymbol{1}}{\boldsymbol{2}\boldsymbol{s}^{\boldsymbol{2}}}\left( \boldsymbol{X}_{\boldsymbol{(j)}}^{\boldsymbol{t}}\boldsymbol{\Sigma}^{\boldsymbol{-1}}\left( \boldsymbol{z} \right)\boldsymbol{+}\frac{\boldsymbol{\mu}_{\boldsymbol{j}}^{\boldsymbol{2}}}{\boldsymbol{\nu}_{\boldsymbol{j}}} \right)^{\boldsymbol{2}} \right\}} \end{matrix}$$

Where $\boldsymbol{z=y-}\boldsymbol{X}_{\left( \boldsymbol{-j} \right)}\boldsymbol{\beta}_{\left( \boldsymbol{-j} \right)}$, $\boldsymbol{s}^{\boldsymbol{2}}\boldsymbol{=}\frac{\boldsymbol{1}}{\boldsymbol{X}_{\boldsymbol{(j)}}^{\boldsymbol{t}}\boldsymbol{\Sigma}^{\boldsymbol{-1}}\boldsymbol{X}_{\left( \boldsymbol{j} \right)}\boldsymbol{+}\frac{\boldsymbol{1}}{\boldsymbol{\nu}_{\boldsymbol{j}}}}$, $\left\{ {\boldsymbol{\mu}_{\boldsymbol{j}}\boldsymbol{,\nu}}_{\boldsymbol{j}} \right\}$ are hyper-parameters from prior distribution of $\boldsymbol{\beta}_{\boldsymbol{j}}$, $\boldsymbol{X}_{\left( \boldsymbol{-j} \right)}$ is the matrix $\boldsymbol{X}$ but without column *j*, $\boldsymbol{\beta}_{\left( \boldsymbol{-j} \right)}$ is the vector $\boldsymbol{\beta}$ without the component j, $\boldsymbol{\Sigma}$ represents the variance structure that corresponds to each model, $\boldsymbol{X}_{\left( \boldsymbol{j} \right)}$ is a vector corresponding to the *j-th* column of matrix $\boldsymbol{X}$.

2.$\boldsymbol{p}\left( \boldsymbol{\gamma}_{\boldsymbol{j}} | \boldsymbol{else} \right)$

$$\begin{matrix} \boldsymbol{p}\left( \boldsymbol{\gamma}_{\boldsymbol{j}}\boldsymbol{=0} | \boldsymbol{else} \right) & \boldsymbol{=} & \frac{\boldsymbol{p}\left( \boldsymbol{\gamma}_{\boldsymbol{j}}\boldsymbol{=0} \right)\boldsymbol{p}\left( \boldsymbol{z} | \boldsymbol{\gamma}_{\boldsymbol{j}}\boldsymbol{=0} \right)}{\boldsymbol{p}\left( \boldsymbol{z} \right)} \\ & \boldsymbol{=} & \frac{\boldsymbol{p}_{\boldsymbol{j}}\boldsymbol{\times p}\left( \boldsymbol{z} | \boldsymbol{\beta}_{\boldsymbol{j}}\boldsymbol{=0} \right)}{\boldsymbol{p}_{\boldsymbol{j}}\boldsymbol{\times p}\left( \boldsymbol{z|}\boldsymbol{\beta}_{\boldsymbol{j}}\boldsymbol{=0} \right)\boldsymbol{+}\left( \boldsymbol{1-}\boldsymbol{p}_{\boldsymbol{j}} \right)\boldsymbol{\times p}\left( \boldsymbol{z} | \boldsymbol{\beta}_{\boldsymbol{j}}\boldsymbol{\neq0} \right)} \\ & \boldsymbol{=} & \frac{\boldsymbol{p}_{\boldsymbol{j}}}{\boldsymbol{p}_{\boldsymbol{j}}\boldsymbol{+}\left( \boldsymbol{1-}\boldsymbol{p}_{\boldsymbol{j}} \right)\boldsymbol{\times BF}}\boldsymbol{=}\boldsymbol{p}_{\boldsymbol{j}}^{\boldsymbol{*}} \end{matrix}$$

Thus, $\boldsymbol{\gamma}_{\boldsymbol{j}}\boldsymbol{|else\sim Bernoulli(1-}\boldsymbol{p}_{\boldsymbol{j}}^{\boldsymbol{*}}\boldsymbol{)}$.

3.$\boldsymbol{p}\left( \boldsymbol{\beta}_{\boldsymbol{j}} | \boldsymbol{else} \right)$

$$\begin{matrix} \boldsymbol{p}\left( \boldsymbol{\beta}_{\boldsymbol{j}}\boldsymbol{,z|}\boldsymbol{\beta}_{\boldsymbol{(-j)}} \right) & \boldsymbol{\propto} & {\boldsymbol{|}\boldsymbol{\Sigma}\boldsymbol{|}}^{\boldsymbol{-1/2}}\boldsymbol{exp}\left\{ \boldsymbol{-}\frac{\boldsymbol{1}}{\boldsymbol{2}}\left( \boldsymbol{z-}\boldsymbol{X}_{\boldsymbol{(j)}}\boldsymbol{\beta}_{\boldsymbol{j}} \right)^{\boldsymbol{t}}\boldsymbol{\Sigma}^{\boldsymbol{-1}}\left( \boldsymbol{z-}\boldsymbol{X}_{\boldsymbol{(j)}}\boldsymbol{\beta}_{\boldsymbol{j}} \right) \right\}\left( \frac{\boldsymbol{1}}{\boldsymbol{\nu}_{\boldsymbol{j}}} \right)^{\boldsymbol{1/2}}\boldsymbol{exp}\left\{ \boldsymbol{-}\frac{\boldsymbol{1}}{\boldsymbol{2}\boldsymbol{\nu}_{\boldsymbol{j}}}\left( \boldsymbol{\beta}_{\boldsymbol{j}}\boldsymbol{-}\boldsymbol{\mu}_{\boldsymbol{j}} \right)^{\boldsymbol{2}} \right\} \\ & \boldsymbol{\propto} & {\boldsymbol{|}\boldsymbol{\Sigma}\boldsymbol{|}}^{\boldsymbol{-1/2}}\left( \frac{\boldsymbol{1}}{\boldsymbol{\nu}_{\boldsymbol{j}}} \right)^{\boldsymbol{1/2}}\boldsymbol{exp}\left\{ \boldsymbol{-}\frac{\boldsymbol{1}}{\boldsymbol{2}}\left( \boldsymbol{z}^{\boldsymbol{t}}\boldsymbol{\Sigma}^{\boldsymbol{-1}}\boldsymbol{z+}\frac{\boldsymbol{\mu}_{\boldsymbol{j}}^{\boldsymbol{2}}}{\boldsymbol{\nu}_{\boldsymbol{j}}} \right)\boldsymbol{+}\frac{\boldsymbol{1}}{\boldsymbol{2}}\boldsymbol{s}^{\boldsymbol{2}}\left( \boldsymbol{X}_{\boldsymbol{(j)}}^{\boldsymbol{t}}\boldsymbol{\Sigma}^{\boldsymbol{-1}}\boldsymbol{z+}\frac{\boldsymbol{\mu}_{\boldsymbol{j}}}{\boldsymbol{\nu}_{\boldsymbol{j}}} \right)^{\boldsymbol{2}} \right\}\boldsymbol{exp}\left\{ \boldsymbol{-}\frac{\boldsymbol{1}}{\boldsymbol{2}\boldsymbol{s}^{\boldsymbol{2}}}\left[ \boldsymbol{\beta}_{\boldsymbol{j}}\boldsymbol{-}\boldsymbol{s}^{\boldsymbol{2}}\left( \boldsymbol{X}_{\boldsymbol{(j)}}^{\boldsymbol{t}}\boldsymbol{\Sigma}^{\boldsymbol{-1}}\boldsymbol{z+}\frac{\boldsymbol{\mu}_{\boldsymbol{j}}}{\boldsymbol{\nu}_{\boldsymbol{j}}} \right) \right]^{\boldsymbol{2}} \right\} \end{matrix}$$

Thus, $\boldsymbol{\beta}_{\boldsymbol{j}}\boldsymbol{|else\sim N}\left( \boldsymbol{s}^{\boldsymbol{2}}\left( \boldsymbol{X}_{\boldsymbol{(j)}}^{\boldsymbol{t}}\boldsymbol{\Sigma}^{\boldsymbol{-1}}\boldsymbol{z+}\frac{\boldsymbol{\mu}_{\boldsymbol{j}}}{\boldsymbol{\nu}_{\boldsymbol{j}}} \right)\boldsymbol{,}\boldsymbol{s}^{\boldsymbol{2}} \right)$.

Algorithm for fitting model (1)

Input: Start with $\boldsymbol{\beta}^{\boldsymbol{(0)}}$, $\boldsymbol{\gamma}^{\boldsymbol{(0)}}$ and $\boldsymbol{\sigma}^{\boldsymbol{2(0)}}$.

Output:$\left( \boldsymbol{\beta}^{\boldsymbol{(1)}}\boldsymbol{,\ldots,}\boldsymbol{\beta}^{\boldsymbol{(100000)}} \right)\boldsymbol{,}\left( \boldsymbol{\gamma}^{\boldsymbol{(1)}}\boldsymbol{,\ldots,}\boldsymbol{\gamma}^{\boldsymbol{(100000)}} \right)$ and $\left( \boldsymbol{\sigma}^{\boldsymbol{2(1)}}\boldsymbol{,\ldots,}\boldsymbol{\sigma}^{\boldsymbol{2(100000)}} \right)$

For i = 1, 2, …,100000 do

For j = 1, 2, …, 100000 do

Compute $\boldsymbol{s}^{\boldsymbol{2}}\boldsymbol{=}\frac{\boldsymbol{1}}{\boldsymbol{X}_{\boldsymbol{(j)}}^{\boldsymbol{t}}\boldsymbol{\sigma}^{\boldsymbol{2(i-1)}}\boldsymbol{X}_{\left( \boldsymbol{j} \right)}\boldsymbol{+}\frac{\boldsymbol{1}}{\boldsymbol{10}}}$

Compute $\boldsymbol{\mu}_{\boldsymbol{\beta}_{\boldsymbol{j}}}\boldsymbol{=}\frac{\boldsymbol{s}^{\boldsymbol{2}}}{\boldsymbol{\sigma}^{\boldsymbol{2(i-1)}}}\left[ \boldsymbol{X}_{\boldsymbol{(j)}}^{\boldsymbol{t}}\left( \boldsymbol{y-}\boldsymbol{X}_{\left( \boldsymbol{-j} \right)}\boldsymbol{\beta}_{\boldsymbol{(-j)}}^{\boldsymbol{(i-1)}} \right) \right]$

Compute $\boldsymbol{BF=}\sqrt{\frac{\boldsymbol{s}^{\boldsymbol{2}}}{\boldsymbol{10}}}\boldsymbol{exp}\left\{ \frac{\boldsymbol{s}^{\boldsymbol{2}}}{\boldsymbol{2}}\left[ \frac{\boldsymbol{X}_{\boldsymbol{(j)}}^{\boldsymbol{t}}\left( \boldsymbol{y-}\boldsymbol{X}_{\left( \boldsymbol{-j} \right)}\boldsymbol{\beta}_{\boldsymbol{(-j)}}^{\boldsymbol{(i-1)}} \right)}{\boldsymbol{\sigma}^{\boldsymbol{2(i-1)}}} \right]^{\boldsymbol{2}} \right\}$

Compute $\boldsymbol{p}_{\boldsymbol{j}}\boldsymbol{=}\frac{\boldsymbol{1}}{\boldsymbol{1+BF}}$

Update $\boldsymbol{\gamma}_{\boldsymbol{j}}^{\boldsymbol{(i)}}\boldsymbol{\sim Bernoulli(1,1-}\boldsymbol{p}_{\boldsymbol{j}}\boldsymbol{)}$

If $\boldsymbol{\gamma}_{\boldsymbol{j}}^{\boldsymbol{(i)}}\boldsymbol{=0}$ then $\boldsymbol{\beta}_{\boldsymbol{j}}^{\boldsymbol{(i)}}\boldsymbol{=0}$ else $\boldsymbol{\beta}_{\boldsymbol{j}}^{\boldsymbol{(i)}}\boldsymbol{\sim N(}\boldsymbol{\mu}_{\boldsymbol{\beta}_{\boldsymbol{j}}}\boldsymbol{,}$ $\boldsymbol{s}^{\boldsymbol{2}}\boldsymbol{)}$

End for

Update $\boldsymbol{\sigma}^{\boldsymbol{2(i)}}\boldsymbol{\sim IG}\left( \boldsymbol{0.01+}\frac{\boldsymbol{n}}{\boldsymbol{2}}\boldsymbol{, 0.01+}\frac{\boldsymbol{1}}{\boldsymbol{2}}\left( \boldsymbol{y-X\beta} \right)^{\boldsymbol{t}}\left( \boldsymbol{y-X\beta} \right) \right)$

End for

Generate m = (50000,50005, …, 99995, 100000)

Compute $\hat{\boldsymbol{\beta}}\boldsymbol{=}\frac{\boldsymbol{1}}{\boldsymbol{10001}}\sum_{\boldsymbol{t\in m}} \boldsymbol{\beta}^{\boldsymbol{(t)}}$

Compute $\hat{\boldsymbol{\gamma}}\boldsymbol{=}\frac{\boldsymbol{1}}{\boldsymbol{10001}}\sum_{\boldsymbol{t\in m}} \boldsymbol{\gamma}^{\boldsymbol{(t)}}$

Compute ${\hat{\boldsymbol{\sigma}}}^{\boldsymbol{2}}\boldsymbol{=}\frac{\boldsymbol{1}}{\boldsymbol{10001}}\sum_{\boldsymbol{t\in m}} \boldsymbol{\sigma}^{\boldsymbol{2(t)}}$

Compute $\boldsymbol{L=}\sum\boldsymbol{log}\left[ \boldsymbol{N(y;X}\hat{\boldsymbol{\beta}}\boldsymbol{,}{\hat{\boldsymbol{\sigma}}}^{\boldsymbol{2}}\boldsymbol{I)} \right]$

Compute $\boldsymbol{P=2}\left\{ \boldsymbol{L-}\frac{\boldsymbol{1}}{\boldsymbol{10001}}\left[ \sum\sum\boldsymbol{log}\left( \boldsymbol{N(y;X}\boldsymbol{\beta}^{\boldsymbol{(t)}}\boldsymbol{,}\boldsymbol{\sigma}^{\boldsymbol{2(t)}}\boldsymbol{I)} \right) \right] \right\}$

Compute $\boldsymbol{DIC=-2(L-P)}$

Algorithm for fitting model (2)

Input: Start with $\boldsymbol{\beta}^{\boldsymbol{(0)}}$, $\boldsymbol{\gamma}^{\boldsymbol{(0)}}$, $\boldsymbol{\sigma}^{\boldsymbol{2(0)}}$, $\boldsymbol{\tau}^{\boldsymbol{2(0)}}$and $\boldsymbol{\phi}^{\boldsymbol{(0)}}$.

Output:$\left( \boldsymbol{\beta}^{\boldsymbol{(1)}}\boldsymbol{,\ldots,}\boldsymbol{\beta}^{\boldsymbol{(100000)}} \right)\boldsymbol{,}\left( \boldsymbol{\gamma}^{\boldsymbol{(1)}}\boldsymbol{,\ldots,}\boldsymbol{\gamma}^{\boldsymbol{(100000)}} \right)$ , $\left( \boldsymbol{\sigma}^{\boldsymbol{2(1)}}\boldsymbol{,\ldots,}\boldsymbol{\sigma}^{\boldsymbol{2(100000)}} \right)$, $\left( \boldsymbol{\tau}^{\boldsymbol{2(1)}}\boldsymbol{,\ldots,}\boldsymbol{\tau}^{\boldsymbol{2(100000)}} \right)$ and $\left( \boldsymbol{\phi}^{\boldsymbol{(1)}}\boldsymbol{,\ldots,}\boldsymbol{\phi}^{\boldsymbol{(100000)}} \right)$

For i = 1, 2, …,100000 do

For j = 1, 2, …, 100000 do

Compute $\boldsymbol{\Sigma}\boldsymbol{=}\frac{\boldsymbol{1}}{\boldsymbol{\tau}^{\boldsymbol{2(i-1)}}}\boldsymbol{I}$

Compute $\boldsymbol{s}^{\boldsymbol{2}}\boldsymbol{=}\frac{\boldsymbol{1}}{\boldsymbol{X}_{\boldsymbol{(j)}}^{\boldsymbol{t}}\boldsymbol{\Sigma}\boldsymbol{X}_{\left( \boldsymbol{j} \right)}\boldsymbol{+}\frac{\boldsymbol{1}}{\boldsymbol{10}}}$

Compute $\boldsymbol{\mu}_{\boldsymbol{\beta}_{\boldsymbol{j}}}\boldsymbol{=}{\boldsymbol{s}^{\boldsymbol{2}}\boldsymbol{X}}_{\boldsymbol{(j)}}^{\boldsymbol{t}}\boldsymbol{\Sigma}\left( \boldsymbol{y-}\boldsymbol{X}_{\left( \boldsymbol{-j} \right)}\boldsymbol{\beta}_{\left( \boldsymbol{-j} \right)}^{\left( \boldsymbol{i-1} \right)}\boldsymbol{-}\boldsymbol{w}^{\boldsymbol{t}} \right)$

Compute $\boldsymbol{BF=}\sqrt{\frac{\boldsymbol{s}^{\boldsymbol{2}}}{\boldsymbol{10}}}\boldsymbol{exp}\left\{ \boldsymbol{-}\frac{\boldsymbol{s}^{\boldsymbol{2}}}{\boldsymbol{2}}\left[ \boldsymbol{X}_{\boldsymbol{(j)}}^{\boldsymbol{t}}\boldsymbol{\Sigma}\left( \boldsymbol{y-}\boldsymbol{X}_{\left( \boldsymbol{-j} \right)}\boldsymbol{\beta}_{\left( \boldsymbol{-j} \right)}^{\left( \boldsymbol{i-1} \right)}\boldsymbol{-}\boldsymbol{w}^{\boldsymbol{t}} \right) \right]^{\boldsymbol{2}} \right\}$

Compute $\boldsymbol{p}_{\boldsymbol{j}}\boldsymbol{=}\frac{\boldsymbol{1}}{\boldsymbol{1+BF}}$

Update $\boldsymbol{\gamma}_{\boldsymbol{j}}^{\boldsymbol{(i)}}\boldsymbol{\sim Bernoulli(1-}\boldsymbol{p}_{\boldsymbol{j}}\boldsymbol{)}$

If $\boldsymbol{\gamma}_{\boldsymbol{j}}^{\boldsymbol{(i)}}\boldsymbol{=0}$ then $\boldsymbol{\beta}_{\boldsymbol{j}}^{\boldsymbol{(i)}}\boldsymbol{=0}$ else $\boldsymbol{\beta}_{\boldsymbol{j}}^{\boldsymbol{(i)}}\boldsymbol{\sim N(}\boldsymbol{\mu}_{\boldsymbol{\beta}_{\boldsymbol{j}}}\boldsymbol{,}$ $\boldsymbol{s}^{\boldsymbol{2}}\boldsymbol{)}$ end if

End for

Update $\boldsymbol{\tau}^{\boldsymbol{2(i)}}\boldsymbol{\sim IG}\left( \boldsymbol{0.01+}\frac{\boldsymbol{n}}{\boldsymbol{2}}\boldsymbol{, 0.01+}\frac{\boldsymbol{1}}{\boldsymbol{2}}\left( \boldsymbol{y-X\beta-}\boldsymbol{w}^{\boldsymbol{t}} \right)^{\boldsymbol{t}}\left( \boldsymbol{y-X\beta-}\boldsymbol{w}^{\boldsymbol{t}} \right) \right)$

Update $\boldsymbol{\sigma}^{\boldsymbol{2(i)}}\boldsymbol{\sim IG}\left( \boldsymbol{0.01+}\frac{\boldsymbol{n}}{\boldsymbol{2}}\boldsymbol{, 0.01+}\frac{\boldsymbol{1}}{\boldsymbol{2}}\boldsymbol{w}^{\boldsymbol{t}}\left[ \boldsymbol{exp}\left( \boldsymbol{-}\boldsymbol{\phi}^{\boldsymbol{(i-1)}}\boldsymbol{D} \right) \right]^{\boldsymbol{-1}}\boldsymbol{w} \right)$

Sample $\boldsymbol{\phi}^{\boldsymbol{*}}\boldsymbol{\sim U(0.2,0.6)}$

Compute $\boldsymbol{\alpha=min}\left( \boldsymbol{1,}\frac{\boldsymbol{N}\left( \boldsymbol{w;0,}\boldsymbol{\tau}^{\boldsymbol{2(i)}}\boldsymbol{exp}\left( \boldsymbol{-}\boldsymbol{\phi}^{\boldsymbol{(i)}}\boldsymbol{D} \right) \right)}{\boldsymbol{N}\left( \boldsymbol{w;0,}\boldsymbol{\tau}^{\boldsymbol{2(i)}}\boldsymbol{exp}\left( \boldsymbol{-}\boldsymbol{\phi}^{\boldsymbol{*}}\boldsymbol{D} \right) \right)} \right)$

Sample $\boldsymbol{u\sim U(0,1)}$

If $\boldsymbol{u}\boldsymbol{\leq}\boldsymbol{\alpha}$ then $\boldsymbol{\phi}^{\boldsymbol{(i)}}\boldsymbol{=}\boldsymbol{\phi}^{\boldsymbol{*}}$

Update $\boldsymbol{w\sim N}\left( \left[ \boldsymbol{\Sigma}\boldsymbol{+}\frac{\left[ \boldsymbol{exp}\left( \boldsymbol{-}\boldsymbol{\phi}^{\boldsymbol{(i)}}\boldsymbol{D} \right) \right]^{\boldsymbol{-1}}}{\boldsymbol{\sigma}^{\boldsymbol{2(i)}}} \right]^{\boldsymbol{-1}}\frac{\boldsymbol{y-X\beta}}{\boldsymbol{\tau}^{\boldsymbol{2(t)}}}\boldsymbol{,}\left[ \boldsymbol{\Sigma}\boldsymbol{+}\frac{\left[ \boldsymbol{exp}\left( \boldsymbol{-}\boldsymbol{\phi}^{\boldsymbol{(i)}}\boldsymbol{D} \right) \right]^{\boldsymbol{-1}}}{\boldsymbol{\sigma}^{\boldsymbol{2(i)}}} \right]^{\boldsymbol{-1}} \right)$

End for

Generate m = (50000,50005, …, 99995, 100000)

Compute $\hat{\boldsymbol{\beta}}\boldsymbol{=}\frac{\boldsymbol{1}}{\boldsymbol{10001}}\sum_{\boldsymbol{t\in m}} \boldsymbol{\beta}^{\boldsymbol{(t)}}$

Compute $\hat{\boldsymbol{\gamma}}\boldsymbol{=}\frac{\boldsymbol{1}}{\boldsymbol{10001}}\sum_{\boldsymbol{t\in m}} \boldsymbol{\gamma}^{\boldsymbol{(t)}}$

Compute ${\hat{\boldsymbol{\sigma}}}^{\boldsymbol{2}}\boldsymbol{=}\frac{\boldsymbol{1}}{\boldsymbol{10001}}\sum_{\boldsymbol{t\in m}} \boldsymbol{\sigma}^{\boldsymbol{2(t)}}$

Compute ${\hat{\boldsymbol{\tau}}}^{\boldsymbol{2}}\boldsymbol{=}\frac{\boldsymbol{1}}{\boldsymbol{10001}}\sum_{\boldsymbol{t\in m}} \boldsymbol{\tau}^{\boldsymbol{2(t)}}$

Compute $\hat{\boldsymbol{\phi}}\boldsymbol{=}\frac{\boldsymbol{1}}{\boldsymbol{10001}}\sum_{\boldsymbol{t\in m}} \boldsymbol{\phi}^{\boldsymbol{(t)}}$

Sample $\boldsymbol{w\sim N}\left( \boldsymbol{0,}{\hat{\boldsymbol{\sigma}}}^{\boldsymbol{2}}\boldsymbol{exp}\left( \boldsymbol{-}\hat{\boldsymbol{\phi}}\boldsymbol{D} \right) \right)$

Compute $\boldsymbol{L=}\sum\boldsymbol{log}\left[ \boldsymbol{N(y;X}\hat{\boldsymbol{\beta}}\boldsymbol{+w,}{\hat{\boldsymbol{\tau}}}^{\boldsymbol{2}}\boldsymbol{I)} \right]$

Compute $\boldsymbol{P=2}\left\{ \boldsymbol{L-}\frac{\boldsymbol{1}}{\boldsymbol{10001}}\left[ \sum\sum\boldsymbol{log}\left( \boldsymbol{N(y;X}\boldsymbol{\beta}^{\boldsymbol{(t)}}\boldsymbol{+w,}{\hat{\boldsymbol{\tau}}}^{\boldsymbol{2(}\boldsymbol{t}\boldsymbol{)}}\boldsymbol{I)} \right) \right] \right\}$

Compute $\boldsymbol{DIC=-2(L-P)}$

Algorithm for fitting model (3)

Input: Start with $\boldsymbol{\beta}^{\boldsymbol{(0)}}$, $\boldsymbol{\gamma}^{\boldsymbol{(0)}}$, $\boldsymbol{\tau}^{\boldsymbol{2(0)}}$and $\boldsymbol{\phi}^{\boldsymbol{(0)}}$.

Output:$\left( \boldsymbol{\beta}^{\boldsymbol{(1)}}\boldsymbol{,\ldots,}\boldsymbol{\beta}^{\boldsymbol{(100000)}} \right)\boldsymbol{,}\left( \boldsymbol{\gamma}^{\boldsymbol{(1)}}\boldsymbol{,\ldots,}\boldsymbol{\gamma}^{\boldsymbol{(100000)}} \right)$, $\left( \boldsymbol{\tau}^{\boldsymbol{2(1)}}\boldsymbol{,\ldots,}\boldsymbol{\tau}^{\boldsymbol{2(100000)}} \right)$ and $\left( \boldsymbol{\phi}^{\boldsymbol{(1)}}\boldsymbol{,\ldots,}\boldsymbol{\phi}^{\boldsymbol{(100000)}} \right)$

For i = 1, 2, …,100000 do

For j = 1, 2, …, 100000 do

Compute$\boldsymbol{\Sigma}\boldsymbol{=}\frac{\boldsymbol{D}_{\boldsymbol{M}}\boldsymbol{-}\boldsymbol{\phi}^{\boldsymbol{(i-1)}}\boldsymbol{M}}{\boldsymbol{\tau}^{\boldsymbol{2(i-1)}}}$

Compute $\boldsymbol{s}^{\boldsymbol{2}}\boldsymbol{=}\frac{\boldsymbol{1}}{\boldsymbol{X}_{\boldsymbol{(j)}}^{\boldsymbol{t}}\boldsymbol{\Sigma}\boldsymbol{X}_{\left( \boldsymbol{j} \right)}\boldsymbol{+}\frac{\boldsymbol{1}}{\boldsymbol{10}}}$

Compute $\boldsymbol{\mu}_{\boldsymbol{\beta}_{\boldsymbol{j}}}\boldsymbol{=}{\boldsymbol{s}^{\boldsymbol{2}}\boldsymbol{X}}_{\boldsymbol{(j)}}^{\boldsymbol{t}}\boldsymbol{\Sigma}\left( \boldsymbol{y-}\boldsymbol{X}_{\left( \boldsymbol{-j} \right)}\boldsymbol{\beta}_{\left( \boldsymbol{-j} \right)}^{\left( \boldsymbol{i-1} \right)} \right)$

Compute $\boldsymbol{BF=}\sqrt{\frac{\boldsymbol{s}^{\boldsymbol{2}}}{\boldsymbol{10}}}\boldsymbol{exp}\left\{ \frac{\boldsymbol{s}^{\boldsymbol{2}}}{\boldsymbol{2}}\left[ \boldsymbol{X}_{\boldsymbol{(j)}}^{\boldsymbol{t}}\boldsymbol{\Sigma}\left( \boldsymbol{y-}\boldsymbol{X}_{\left( \boldsymbol{-j} \right)}\boldsymbol{\beta}_{\left( \boldsymbol{-j} \right)}^{\left( \boldsymbol{i-1} \right)} \right) \right]^{\boldsymbol{2}} \right\}$

Compute $\boldsymbol{p}_{\boldsymbol{j}}\boldsymbol{=}\frac{\boldsymbol{1}}{\boldsymbol{1+BF}}$

Update $\boldsymbol{\gamma}_{\boldsymbol{j}}^{\boldsymbol{(i)}}\boldsymbol{\sim Bin(1,1-}\boldsymbol{p}_{\boldsymbol{j}}\boldsymbol{)}$

If $\boldsymbol{\gamma}_{\boldsymbol{j}}^{\boldsymbol{(i)}}\boldsymbol{=0}$ then $\boldsymbol{\beta}_{\boldsymbol{j}}^{\boldsymbol{(i)}}\boldsymbol{=0}$ else $\boldsymbol{\beta}_{\boldsymbol{j}}^{\boldsymbol{(i)}}\boldsymbol{\sim N(}\boldsymbol{\mu}_{\boldsymbol{\beta}_{\boldsymbol{j}}}\boldsymbol{,}$ $\boldsymbol{s}^{\boldsymbol{2}}\boldsymbol{)}$ end if

End for

Update $\boldsymbol{\tau}^{\boldsymbol{2(i)}}\boldsymbol{\sim IG}\left( \boldsymbol{0.01+}\frac{\boldsymbol{n}}{\boldsymbol{2}}\boldsymbol{, 0.01+}\frac{\boldsymbol{1}}{\boldsymbol{2}}\left( \boldsymbol{y-X\beta} \right)^{\boldsymbol{t}}\left( \boldsymbol{D}_{\boldsymbol{M}}\boldsymbol{-}\boldsymbol{\phi}^{\boldsymbol{(i-1)}}\boldsymbol{M} \right)\left( \boldsymbol{y-X\beta} \right) \right)$

Compute $\boldsymbol{\phi}^{\boldsymbol{*}}\boldsymbol{\sim N}\left( \boldsymbol{\phi}^{\boldsymbol{(i-1)}}\boldsymbol{;0.25,}\boldsymbol{\lambda}_{\boldsymbol{1}}\boldsymbol{,}\boldsymbol{\lambda}_{\boldsymbol{n}} \right)$

Compute $\boldsymbol{\alpha=min}\left( \boldsymbol{1,}\frac{\boldsymbol{N}\left( \boldsymbol{\phi}^{\boldsymbol{(i-1)}}\boldsymbol{;}\boldsymbol{\phi}^{\boldsymbol{*}}\boldsymbol{,1,}\boldsymbol{\lambda}_{\boldsymbol{1}}\boldsymbol{,}\boldsymbol{\lambda}_{\boldsymbol{n}} \right)}{\boldsymbol{N}\left( {\boldsymbol{\phi}^{\boldsymbol{*}}\boldsymbol{;\phi}}^{\boldsymbol{(i-1)}}\boldsymbol{,1,}\boldsymbol{\lambda}_{\boldsymbol{1}}\boldsymbol{,}\boldsymbol{\lambda}_{\boldsymbol{n}} \right)} \right)$

Compute $\boldsymbol{u\sim U(0,1)}$

If $\boldsymbol{u}\boldsymbol{\leq}\boldsymbol{\alpha}$ then update $\boldsymbol{\phi}^{\boldsymbol{(i)}}\boldsymbol{=}\boldsymbol{\phi}^{\boldsymbol{*}}$ end if

End for

Generate m = (50000,50005, …, 99995, 100000)

Compute $\hat{\boldsymbol{\beta}}\boldsymbol{=}\frac{\boldsymbol{1}}{\boldsymbol{10001}}\sum_{\boldsymbol{t\in m}} \boldsymbol{\beta}^{\boldsymbol{(t)}}$

Compute $\hat{\boldsymbol{\gamma}}\boldsymbol{=}\frac{\boldsymbol{1}}{\boldsymbol{10001}}\sum_{\boldsymbol{t\in m}} \boldsymbol{\gamma}^{\boldsymbol{(t)}}$

Compute ${\hat{\boldsymbol{\tau}}}^{\boldsymbol{2}}\boldsymbol{=}\frac{\boldsymbol{1}}{\boldsymbol{10001}}\sum_{\boldsymbol{t\in m}} \boldsymbol{\tau}^{\boldsymbol{2(t)}}$

Compute $\hat{\boldsymbol{\phi}}\boldsymbol{=}\frac{\boldsymbol{1}}{\boldsymbol{10001}}\sum_{\boldsymbol{t\in m}} \boldsymbol{\phi}^{\boldsymbol{(t)}}$

Compute $\boldsymbol{L=}\sum\boldsymbol{log}\left[ \boldsymbol{N}\left( \boldsymbol{y;X}\hat{\boldsymbol{\beta}}\boldsymbol{,}{\hat{\boldsymbol{\tau}}}^{\boldsymbol{2}}\left( \boldsymbol{D}_{\boldsymbol{M}}\boldsymbol{-}\hat{\boldsymbol{\phi}}\boldsymbol{M} \right)^{\boldsymbol{-1}} \right) \right]$

Compute $\boldsymbol{P=2}\left\{ \boldsymbol{L-}\frac{\boldsymbol{1}}{\boldsymbol{10001}}\left[ \sum\sum\boldsymbol{log}\left( \boldsymbol{N}\left( \boldsymbol{y;X}\boldsymbol{\beta}^{\boldsymbol{(t)}}\boldsymbol{,}{\hat{\boldsymbol{\tau}}}^{\boldsymbol{2}}\left( \boldsymbol{D}_{\boldsymbol{M}}\boldsymbol{-}\hat{\boldsymbol{\phi}}\boldsymbol{M} \right)^{\boldsymbol{-1}} \right) \right) \right] \right\}$

Compute $\boldsymbol{DIC=-2(L-P)}$
