# Supplementary material for: Bayesian modelling of phosphorus content in wheat grain using hyperspectral reflectance data
Source: Plant Methods. 2023 Jan 20;19:6. doi: 10.1186/s13007-023-00980-9 (PMC9854047; doi:10.1186/s13007-023-00980-9)
Supplement: Supplementary file 3 — Additional file 3: R codes. [file 13007_2023_980_MOESM3_ESM.docx]

**Information objects description.**

ParaServerX.RData: Contain in” X” the independent variables, and “y” is the response variable for year 2010.

ParaServerY.RData: Contain in “X” the independent variables, and “y” is the response variable for year 2011.

ParaServerZ.RData: Contain in “X” the independent variables, and “y” is the response variable for year 2012.

Muestras.RData: Contain in “muestra80train20test” the testing train used for the cross validation.

Sigma2010.RData: Contain in Dm the neighborhood matrix.

DataXYZ.csv: The information about row and column for each plot.

**Code in R for SCE model**

#Libraries used

library(MCMCpack)

library(truncnorm)

library(mvtnorm)

#Function for calculate DIC

logLikelihood = function(y, mu, sigma2) {

mum=X%*%mu

sum( dnorm(t(y), mum,sigma2, log=TRUE) )

}

calculateDIC = function(y, mu_post, sigma2_post, llFun) {

#Calculate L

theta_hat = apply(mu_post, 2, mean)

sigma_hat = mean(sigma2_post)

L = llFun(y, theta_hat, sigma_hat)

#Calculate P

S = seq(50000, 100000,by=5)

llSum = 0

for (s in S) {

llSum = llSum + llFun(y, mu_post[s,], sigma2_post[s,])

if( s %% 100 == 0 ) cat("iter:",s,"\n")

}

P = 2 * (L - (1 / length(S) * llSum))

#Calculate DIC

DIC = -2 * (L - P)

list(DIC=DIC, P=P, L=L)

}

#Read data

setwd("url")

load("ParaServerX.RData")

#Started values

n<-dim(X)[[1]]

dimX<-dim(X)[[2]]

Beta<-matrix(0, nrow=dimX, ncol=1)

Beta.pos<-matrix(0,nrow=niteration,ncol=dimX)

Delta.pos<-matrix(0,nrow=niteration,ncol=dimX)

Sigma.pos<-matrix(0,nrow=niteration,ncol=1)

vj=10

delta<-matrix(0,ncol=1,nrow=dimX)

sigma2=1

alphasigma=.01; betasigma=.01

niteration<<-100000

#Start Gibbs algorithm

for (i in 1:niteration){

for(j in 1:dimX){

s2=1/(crossprod(X[,j])/sigma2+1/vj)

BetaMu=s2*crossprod(X[,j], ystar-X[,-j]%*%Beta[-j])/sigma2

BF=sqrt(s2/vj)*exp(0.5*s2*(crossprod(X[,j],ystar-X[,-j]%*%Beta[-j])/sigma2)^2)

p.j=(1+BF)^-1

delta[j]=rbinom(1,1,1-p.j)

if(delta[j]==0) {

Beta[j]=0

}

else {

Beta[j]=rnorm(1,BetaMu,sqrt(s2))

}

}

Beta.pos[i,]=Beta

sigma2=rinvgamma(1,shape=alphasigma+n/2,scale=betasigma+crossprod(ystar-X%*%Beta)/2)

Sigma.pos[i]=sigma2

Delta.pos[i,]=delta

if( i %% 100 == 0 ) cat("iter:",i,"\n")

}

#Calculate el DIC

cDIC=calculateDIC(ystar,Beta.pos,Sigma.pos,logLikelihood)

#To select variables with probability bigger tan 0.6

forby=seq(50000, 100000,by=5)

RowN=colnames(X)

probBB=colMeans(Delta.pos[forby,])>.6

varselBB=which(colnames(X)%in%RowN[colMeans(Delta.pos[forby,])>.6])

#To select the more probable model

Delta.use=as.data.frame(Delta.pos[forby,])

compDelta=matrix(0,dim(Delta.use)[1],1)

for(i in 1:dim(Delta.use)[1]){

compDelta[i]=paste(Delta.pos[i,],collapse="")

}

lenModTot=length(unique(compDelta))

ModTot=unique(compDelta)

freqDelta=matrix(0,lenModTot,1)

for (i in 1:lenModTot){

freqDelta[i,]=length(which(compDelta==ModTot[i,]))/dim(Delta.use)[1]

}

bestmodel=ModTot[which(freqDelta==max(freqDelta))]

varselavg=which(unlist(strsplit(bestmodel,""))=="1")

#Start cross-validation

betasam=list()

sigmasam=list()

corsam=list()

yobsam=list()

ypredsam=list()

load("muestras.RData")

ystarall=ystar

Xall=X

for (k in 1:5){

yobs=ystarall[muestra80train20test[,k],]

ystar=ystarall[-muestra80train20test[,k],]

X=Xall[-muestra80train20test[,k],]

niteration<<-100000

n<-dim(X)[[1]]

dimX<-dim(X)[[2]]

Beta<-matrix(0, nrow=dimX, ncol=1)

Beta.pos<-matrix(0,nrow=niteration,ncol=dimX)

Delta.pos<-matrix(0,nrow=niteration,ncol=dimX)

Sigma.pos<-matrix(0,nrow=niteration,ncol=1)

vj=10

delta<-matrix(0,ncol=1,nrow=dimX)

sigma2=1

alphasigma=.01; betasigma=.01

for (i in 1:niteration){

for(j in 1:dimX){

s2=1/(crossprod(X[,j])/sigma2+1/vj)

BetaMu=s2*crossprod(X[,j], ystar-X[,-j]%*%Beta[-j])/sigma2

BF=sqrt(s2/vj)*exp(0.5*s2*(crossprod(X[,j],ystar-X[,-j]%*%Beta[-j])/sigma2)^2)

p.j=(1+BF)^-1

delta[j]=rbinom(1,1,1-p.j)

if(delta[j]==0) {

Beta[j]=0

}

else {

Beta[j]=rnorm(1,BetaMu,sqrt(s2))

}

}

Beta.pos[i,]=Beta

sigma2=rinvgamma(1,shape=alphasigma+n/2,scale=betasigma+crossprod(ystar-X%*%Beta)/2)

Sigma.pos[i]=sigma2

Delta.pos[i,]=delta

if( i %% 100 == 0 ) cat("iter:",i,"\n")

}

forby=seq(50000, 100000,by=5)

mupred=Xall[muestra80train20test[,k],]%*%apply(Beta.pos[forby,],2,mean)

sigpred=sqrt(mean(Sigma.pos[forby,]))

ypred=rnorm(25,mupred,sigpred)

betasam[[k]]=apply(Beta.pos[forby,],2,mean)

sigmasam[[k]]=mean(Sigma.pos[forby,])

corsam[[k]]=cor(yobs,ypred)

ypredsam[[k]]=ypred

yobsam[[k]]=yobs

**Code in R for geospatial model**

#Libraries used

library(MCMCpack)

library(mvtnorm)

library(truncnorm)

#Function for calculate DIC

logLikelihood = function(y, mu, sigma2, phi,tau2) {

D=as.matrix(dist(coords))

R=exp(-phi*D)

w=rmvnorm(1, rep(0,126), tau2*R)

mum=X%*%mu+t(w)

varm=sigma2*diag(n)

sum( dmvnorm(t(y), mum, varm, log=TRUE) )

}

calculateDIC = function(y, mu_post, sigma2_post, phi_post, tau2_post,llFun) {

#Calculate L

theta_hat = apply(mu_post, 2, mean)

sigma_hat = mean(sigma2_post)

phi_hat =mean(phi_post)

tau2_hat =mean(tau2_post)

L = llFun(y, theta_hat, sigma_hat, phi_hat,tau2_hat)

#Calculate P

S = seq(50000, 100000,by=5)

llSum = 0

for (s in S) {

llSum = llSum + llFun(y, mu_post[s,], sigma2_post[s,], phi_post[s,],tau2_post[s,])

if( s %% 100 == 0 ) cat("iter:",s,"\n")

}

P = 2 * (L - (1 / length(S) * llSum))

#Calculate DIC

DIC = -2 * (L - P)

list(DIC=DIC, P=P, L=L)

}

#Read data

setwd("url")

load("ParaServerX.RData")

load("sigma2010.RData")

DataXYZN <- read.csv("DataXYZ.csv")

coords <- cbind(as.numeric(DataXYZN$Column[1:126]),as.numeric(DataXYZN$Row[1:126]))

#Start values

n<<-dim(X)[[1]]

dimX<<-dim(X)[[2]]

Beta<<-matrix(0, nrow=dimX, ncol=1)

Beta.pos<<-matrix(0,nrow=niteration,ncol=dimX)

Delta.pos<<-matrix(0,nrow=niteration,ncol=dimX)

sigma.pos<<-matrix(0.01,nrow=niteration+1,ncol=1)

tau2.pos<<-matrix(0.01,nrow=niteration+1,ncol=1)

phi.pos<<-matrix(0.2,nrow=niteration+1,ncol=1)

vj<<-10

sigma2=1

tau2=1

phi=runif(1,0,1)

delta<<-matrix(0,ncol=1,nrow=dimX)

alphasigma<<-1; betasigma<<-1

D=as.matrix(dist(coords))

R=exp(-0.3*D)

w=rmvnorm(1, rep(0,dim(R)[1]), 0.1*R)

sigmainv=(1/sigma2)*diag(n)

niteration<<-100000

#Start Gibbs algorithm

for (i in 1:niteration){

for(j in 1:dimX){

s2=1/(t(X[,j])%*%sigmainv%*%X[,j]+1/vj)

BetaMu=s2*(t(X[,j])%*%sigmainv%*%(ystar-(X[,-j]%*%Beta[-j])-t(w)))

BF=sqrt(s2/vj)*exp(0.5*s2*(t(X[,j])%*%sigmainv%*%(ystar-(X[,-j]%*%Beta[-j])-t(w)))^2)

p.j=(1+BF)^-1

delta[j]=rbinom(1,1,1-p.j)

if(delta[j]==0) {

Beta[j]=0

}

else {

Beta[j]=rnorm(1,BetaMu,sqrt(s2))

}

}

Beta.pos[i,]=Beta

tau2=rinvgamma(1,shape=alphasigma+(n/2),scale=betasigma+((w)%*%solve(exp(-phi*D))%*%t(w))/2)

sigma2=rinvgamma(1,shape=alphasigma+(n/2),

scale=betasigma+(t(ystar-(X%*%Beta)-t(w))%*%(ystar-(X%*%Beta)-t(w)))/2)

qphi=runif(1,0,1)

change=dmvnorm(w,rep(0,n),tau2*exp(-phi*D))/ (dmvnorm(w,rep(0,n),tau2*exp(-qphi*D)))

alpha=min(1,change)

if (runif(1)<=alpha) phi=qphi

inv1=solve((1/sigma2)*diag(n)+(1/tau2)*solve(exp(-phi*D)))

w=rmvnorm(1, inv1%*%((1/sigma2)*(ystar-X%*%Beta)), inv1)

sigmainv=(1/sigma2)*diag(n)

sigma.pos[i]=sigma2

tau2.pos[i]=tau2

phi.pos[i]=phi

Delta.pos[i,]=delta

if( i %% 100 == 0 ) cat("iter:",i,"\n")

}

#Calculate DIC

cDIC=calculateDIC(ystar,Beta.pos,sigma.pos,phi.pos,tau2.pos,logLikelihood)

#To select variables with probability bigger than 0.6

forby=seq(50000, 100000,by=5)

RowN=colnames(X)

probBB=colMeans(Delta.pos[forby,])>.6

varselBB=which(colnames(X)%in%RowN[colMeans(Delta.pos[forby,])>.6])

#To select the most probable model

Delta.use=as.data.frame(Delta.pos[forby,])

compDelta=matrix(0,dim(Delta.use)[1],1)

for(i in 1:dim(Delta.use)[1]){

compDelta[i]=paste(Delta.pos[i,],collapse="")

}

lenModTot=length(unique(compDelta))

ModTot=unique(compDelta)

freqDelta=matrix(0,lenModTot,1)

for (i in 1:lenModTot){

freqDelta[i,]=length(which(compDelta==ModTot[i,]))/dim(Delta.use)[1]

}

bestmodel=ModTot[which(freqDelta==max(freqDelta))]

varselavg=which(unlist(strsplit(bestmodel,""))=="1")

#Start cross-validation

betasam=list()

sigmasam=list()

phisam=list()

tau2sam=list()

corsam=list()

yobsam=list()

ypredsam=list()

load("muestras.RData")

Xall=X

ystarall=ystar

for (k in 1:5){

yobs=ystarall[muestra80train20test[,k],]

ystar=ystarall[-muestra80train20test[,k],]

X=Xall[-muestra80train20test[,k],]

niteration<<-100000

n<<-dim(X)[[1]]

dimX<<-dim(X)[[2]]

Beta<<-matrix(0, nrow=dimX, ncol=1)

Beta.pos<<-matrix(0,nrow=niteration,ncol=dimX)

Delta.pos<<-matrix(0,nrow=niteration,ncol=dimX)

sigma.pos<<-matrix(0.01,nrow=niteration+1,ncol=1)

tau2.pos<<-matrix(0.01,nrow=niteration+1,ncol=1)

phi.pos<<-matrix(0.2,nrow=niteration+1,ncol=1)

vj<<-10

sigma2=1

tau2=1

phi=runif(1,0,1)

delta<<-matrix(0,ncol=1,nrow=dimX)

alphasigma<<-1; betasigma<<-1

D=as.matrix(dist(coords[-muestra80train20test[,k],]))

R=exp(-0.3*D)

w=rmvnorm(1, rep(0,dim(R)[1]), 0.1*R)

sigmainv=(1/sigma2)*diag(n)

for (i in 1:niteration){

for(j in 1:dimX){

s2=1/(t(X[,j])%*%sigmainv%*%X[,j]+1/vj)

BetaMu=s2*(t(X[,j])%*%sigmainv%*%(ystar-(X[,-j]%*%Beta[-j])-t(w)))

BF=sqrt(s2/vj)*exp(0.5*s2*(t(X[,j])%*%sigmainv%*%(ystar-(X[,-j]%*%Beta[-j])-t(w)))^2)

p.j=(1+BF)^-1

delta[j]=rbinom(1,1,1-p.j)

if(delta[j]==0) {

Beta[j]=0

}

else {

Beta[j]=rnorm(1,BetaMu,sqrt(s2))

}

}

Beta.pos[i,]=Beta

tau2=rinvgamma(1,shape=alphasigma+(n/2),scale=betasigma+((w)%*%solve(exp(-phi*D))%*%t(w))/2)

sigma2=rinvgamma(1,shape=alphasigma+(n/2),

scale=betasigma+(t(ystar-(X%*%Beta)-t(w))%*%(ystar-(X%*%Beta)-t(w)))/2)

qphi=runif(1,0,1)

change=dmvnorm(w,rep(0,n),tau2*exp(-phi*D))/ (dmvnorm(w,rep(0,n),tau2*exp(-qphi*D)))

alpha=min(1,change)

if (runif(1)<=alpha) phi=qphi

inv1=solve((1/sigma2)*diag(n)+(1/tau2)*solve(exp(-phi*D)))

w=rmvnorm(1, inv1%*%((1/sigma2)*(ystar-X%*%Beta)), inv1)

sigmainv=(1/sigma2)*diag(n)

sigma.pos[i]=sigma2

tau2.pos[i]=tau2

phi.pos[i]=phi

Delta.pos[i,]=delta

if( i %% 100 == 0 ) cat("iter:",i,"\n")

}

forby=seq(50000,100000,5)

betasam[[k]]=apply(Beta.pos[forby,],2,mean)

sigmasam[[k]]=mean(sigma.pos[forby,])

phisam[[k]]=mean(phi.pos[forby,])

tau2sam[[k]]=mean(tau2.pos[forby,])

Sall=tau2sam[[k]]*exp(-phisam[[k]]*as.matrix(dist(coords)))+diag(sigmasam[[k]],126)

SComp=rbind(cbind(Sall[muestra80train20test[,k],muestra80train20test[,k]],

Sall[muestra80train20test[,k],-muestra80train20test[,k]]),

cbind(Sall[-muestra80train20test[,k],muestra80train20test[,k]],

Sall[-muestra80train20test[,k],-muestra80train20test[,k]]))

mupred=(Xall[muestra80train20test[,k],]%*%betasam[[k]])+

(SComp[1:25,26:126]%*%solve(SComp[26:126,26:126]))%*%(ystarall[-muestra80train20test[,k],]-

Xall[-muestra80train20test[,k],]%*%betasam[[k]])

varpred=SComp[1:25,1:25]-(SComp[1:25,26:126]%*%solve(SComp[26:126,26:126])%*%SComp[26:126,1:25])

ypred=t(rmvnorm(1,mupred,varpred))

corsam[[k]]=cor(yobs,ypred)

ypredsam[[k]]=ypred

yobsam[[k]]=yobs

**Code in R for CAR model**

#Libraries used

library(MCMCpack)

library(truncnorm)

library(mvtnorm)

#Function for calculate DIC

logLikelihood = function(y, mu, sigma2, phi) {

mum=X%*%mu

varm=sigma2*solve(Dw-phi*W)

sum( dmvnorm(t(y), mum, varm, log=TRUE) )

}

calculateDIC = function(y, mu_post, sigma2_post, phi_post, llFun) {

#Calculate L

theta_hat = apply(mu_post, 2, mean)

sigma_hat = mean(sigma2_post)

phi_hat =mean(phi_post)

L = llFun(y, theta_hat, sigma_hat, phi_hat)

#Calculate P

S = seq(50000, 100000,by=5)

llSum = 0

for (s in S) {

llSum = llSum + llFun(y, mu_post[s,], sigma2_post[s,], phi_post[s,])

if( s %% 100 == 0 ) cat("iter:",s,"\n")

}

P = 2 * (L - (1 / length(S) * llSum))

#Calculate DIC

DIC = -2 * (L - P)

list(DIC=DIC, P=P, L=L)

}

#Read data

setwd(" ")

load("ParaServerY.RData")

load("sigma2010.RData")

#Start values

n<<-dim(X)[[1]]

dimX<<-dim(X)[[2]]

Beta<<-matrix(0, nrow=dimX, ncol=1)

Beta.pos<<-matrix(0,nrow=niteration,ncol=dimX)

Delta.pos<<-matrix(0,nrow=niteration,ncol=dimX)

sigma.pos<<-matrix(0.0002,nrow=niteration,ncol=1)

phi.pos<<-matrix(0.0002,nrow=niteration,ncol=1)

vj<<-10

sigma2<<-1

delta<<-matrix(0,ncol=1,nrow=dimX)

alphasigma<<-1; betasigma<<-1

Dw=diag(rowSums(Dm))

W=Dm

Weig=solve(Dw)^(1/2)%*%W%*%solve(Dw)^(1/2)

lambdas=1/eigen(Weig,only.values = T)$values

lambda.1=lambdas[1]

lambda.n=lambdas[n]

phi=runif(1,lambda.n,lambda.1)

sigmainv=(Dw-phi*W)/sigma2

niteration<<-100000

#Start Gibbs algorithm

for (i in 1:niteration){

for(j in 1:dimX){

s2=1/(t(X[,j])%*%sigmainv%*%X[,j]+1/vj)

if(dimX!=2){

BetaMu=s2*t(X[,j])%*%sigmainv%*%(ystar-X[,-j]%*%Beta[-j])

BF=sqrt(s2/vj)*exp(0.5*s2*(t(X[,j])%*%sigmainv%*%(ystar-X[,-j]%*%Beta[-j]))^2)

}else{

BetaMu=s2*t(X[,j])%*%sigmainv%*%(ystar-X[,-j]*Beta[-j])

BF=sqrt(s2/vj)*exp(0.5*s2*(t(X[,j])%*%sigmainv%*%(ystar-X[,-j]*Beta[-j]))^2)

}

p.j=(1+BF)^-1

delta[j]=rbinom(1,1,1-p.j)

if(delta[j]==0) {

Beta[j]=0

}

else {

Beta[j]=rnorm(1,BetaMu,sqrt(s2))

}

}

Beta.pos[i,]=Beta

sigma2=rinvgamma(1,shape=alphasigma+(n/2),

scale=betasigma+(t(ystar-X%*%Beta)%*%(Dw-phi*W)%*%(ystar-X%*%Beta))/2)

phinew=rtruncnorm(1,lambda.n,lambda.1,phi,0.25)

r1=as.numeric((1/2)*(determinant((Dw-phinew*W),logarithm =T)$modulus-

determinant((Dw-phi*W),logarithm=T)$modulus)-

(1/(2*sigma2)*t(ystar-X%*%Beta)%*%((Dw-phinew*W)-(Dw-phi*W))%*%(ystar-X%*%Beta))) r3=dtruncnorm(phi,lambda.n,lambda.1,phinew,1)/dtruncnorm(phinew,lambda.n,lambda.1,phi,1)

alpha=log(r3)+r1

if (log(runif(1))<=alpha){phi=phinew}

sigmainv=(Dw-phi*W)/sigma2

sigma.pos[i]=sigma2

phi.pos[i]=phi

Delta.pos[i,]=delta

if( i %% 100 == 0 ) cat("iter:",i,"\n")

}

#Calculate DIC

cDIC=calculateDIC(ystar,Beta.pos,sigma.pos,phi.pos,logLikelihood)

#To select variables with probability bigger than 0.6

forby=seq(10000, 25000,by=5)

RowN=colnames(X)

probBB=colMeans(Delta.pos[forby,])>.6

varselBB=which(colnames(X)%in%RowN[colMeans(Delta.pos[forby,])>.6])

##To select the most probable model

Delta.use=as.data.frame(Delta.pos[forby,])

compDelta=matrix(0,dim(Delta.use)[1],1)

for(i in 1:dim(Delta.use)[1]){

compDelta[i]=paste(Delta.pos[i,],collapse="")

}

lenModTot=length(unique(compDelta))

ModTot=unique(compDelta)

freqDelta=matrix(0,lenModTot,1)

for (i in 1:lenModTot){

freqDelta[i,]=length(which(compDelta==ModTot[i,]))/dim(Delta.use)[1]

}

bestmodel=ModTot[which(freqDelta==max(freqDelta))]

varselavg=which(unlist(strsplit(bestmodel,""))=="1")

#Start cross-validation

betasam=list()

sigmasam=list()

corsam=list()

phisam=list()

yobsam=list()

ypredsam=list()

load("muestras.RData")

ystarall=ystar

coords <- cbind(as.numeric(DataXYZN$Column[1:126]),as.numeric(DataXYZN$Row[1:126]),1:126)

Xall=X

Dmall=Dm

for (k in 1:5){

yobs=ystarall[muestra80train20test[,k],]

ystar=ystarall[-muestra80train20test[,k],]

X=Xall[-muestra80train20test[,k],]

niteration<<-100000

n<<-dim(X)[[1]]

dimX<<-dim(X)[[2]]

Beta<<-matrix(0, nrow=dimX, ncol=1)

Beta.pos<<-matrix(0,nrow=niteration,ncol=dimX)

Delta.pos<<-matrix(0,nrow=niteration,ncol=dimX)

sigma.pos<<-matrix(0.0002,nrow=niteration,ncol=1)

phi.pos<<-matrix(0.0002,nrow=niteration,ncol=1)

vj<<-10

sigma2<<-1

delta<<-matrix(0,ncol=1,nrow=dimX)

alphasigma<<-1; betasigma<<-1

coorduse=coords

coorduse[muestra80train20test[,k],3]=NA

Dm=calcNB(as.data.frame(coorduse))

Dw=diag(rowSums(Dm))

W=Dm

Weig=solve(Dw)^(1/2)%*%W%*%solve(Dw)^(1/2)

lambdas=1/eigen(Weig,only.values = T)$values

lambda.1=lambdas[1]

lambda.n=lambdas[n]

phi=runif(1,lambda.n,lambda.1)

sigmainv=(Dw-phi*W)/sigma2

for (i in 1:niteration){

for(j in 1:dimX){

s2=1/(t(X[,j])%*%sigmainv%*%X[,j]+1/vj)

if(dimX!=2){

BetaMu=s2*t(X[,j])%*%sigmainv%*%(ystar-X[,-j]%*%Beta[-j])

BF=sqrt(s2/vj)*exp(0.5*s2*(t(X[,j])%*%sigmainv%*%(ystar-X[,-j]%*%Beta[-j]))^2)

}else{

BetaMu=s2*t(X[,j])%*%sigmainv%*%(ystar-X[,-j]*Beta[-j])

BF=sqrt(s2/vj)*exp(0.5*s2*(t(X[,j])%*%sigmainv%*%(ystar-X[,-j]*Beta[-j]))^2)

}

p.j=(1+BF)^-1

delta[j]=rbinom(1,1,1-p.j)

if(delta[j]==0) {

Beta[j]=0

}

else {

Beta[j]=rnorm(1,BetaMu,sqrt(s2))

}

}

Beta.pos[i,]=Beta

sigma2=rinvgamma(1,shape=alphasigma+(n/2),

scale=betasigma+(t(ystar-X%*%Beta)%*%(Dw-phi*W)%*%(ystar-X%*%Beta))/2)

phinew=rtruncnorm(1,lambda.n,lambda.1,phi,0.25)

r1=as.numeric((1/2)*(determinant((Dw-phinew*W),logarithm =T)$modulus-

determinant((Dw-phi*W),logarithm=T)$modulus)-

(1/(2*sigma2)*t(ystar-X%*%Beta)%*%((Dw-phinew*W)-(Dw-phi*W))%*%(ystar-X%*%Beta))) r3=dtruncnorm(phi,lambda.n,lambda.1,phinew,1)/dtruncnorm(phinew,lambda.n,lambda.1,phi,1)

alpha=log(r3)+r1

if (log(runif(1))<=alpha){phi=phinew}

sigmainv=(Dw-phi*W)/sigma2

sigma.pos[i]=sigma2

phi.pos[i]=phi

Delta.pos[i,]=delta

if( i %% 100 == 0 ) cat("iter:",i,"\n")

}

forby=seq(50000, 100000,by=5)

Wallpred=rbind(cbind(Dmall[muestra80train20test[,k],muestra80train20test[,k]],

Dmall[muestra80train20test[,k],-muestra80train20test[,k]]),

cbind(Dmall[-muestra80train20test[,k],muestra80train20test[,k]],

Dmall[-muestra80train20test[,k],-muestra80train20test[,k]]))

DwAllpred=diag(rowSums(Wallpred))

SComp=mean(sigma.pos[forby,])*solve(DwAllpred-mean(phi.pos[forby,])*Wallpred)

mupred=(Xall[muestra80train20test[,k],]%*%apply(Beta.pos[forby,],2,mean))+

(SComp[1:25,26:126]%*%solve(SComp[26:126,26:126]))%*%(ystarall[-muestra80train20test[,k],]-

Xall[-muestra80train20test[,k],]%*%apply(Beta.pos[forby,],2,mean))

varpred=SComp[1:25,1:25]-(SComp[1:25,26:126]%*%solve(SComp[26:126,26:126])%*%SComp[26:126,1:25])

ypred=t(rmvnorm(1,mupred,varpred))

betasam[[k]]=apply(Beta.pos[forby,],2,mean)

sigmasam[[k]]=mean(sigma.pos[forby,])

phisam[[k]]=mean(phi.pos[forby,])

corsam[[k]]=cor(yobs,ypred)

ypredsam[[k]]=ypred

yobsam[[k]]=yobs
